# Concerted regulation of skeletal muscle metabolism and contractile properties by the orphan nuclear receptor Nr2f6

**DOI:** 10.1002/jcsm.13480

**Published:** 2024-04-29

**Authors:** Dimitrius Santiago P. S. F. Guimarães, Ninon M. F. Barrios, André Gustavo de Oliveira, David Rizo‐Roca, Maxence Jollet, Jonathon A.B. Smith, Thiago R. Araujo, Marcos Vinicius da Cruz, Emilio Marconato, Sandro M. Hirabara, André S. Vieira, Anna Krook, Juleen R. Zierath, Leonardo R. Silveira

**Affiliations:** ^1^ Department of Physiology and Pharmacology Karolinska Institutet Stockholm Sweden; ^2^ Department of Structural and Functional Biology University of Campinas Campinas Brazil; ^3^ Department of Molecular Medicine and Surgery Karolinska Institutet Stockholm Sweden; ^4^ Interdisciplinary Post‐Graduate Program in Health Sciences Cruzeiro do Sul University São Paulo Brazil

**Keywords:** Metabolism, Muscle atrophy, Nr2f6, Skeletal muscle, Transcription

## Abstract

**Background:**

The maintenance of skeletal muscle plasticity upon changes in the environment, nutrient supply, and exercise depends on regulatory mechanisms that couple structural and metabolic adaptations. The mechanisms that interconnect both processes at the transcriptional level remain underexplored. Nr2f6, a nuclear receptor, regulates metabolism and cell differentiation in peripheral tissues. However, its role in the skeletal muscle is still elusive. Here, we aimed to investigate the effects of Nr2f6 modulation on muscle biology *in vivo* and *in vitro*.

**Methods:**

Global RNA‐seq was performed in Nr2f6 knockdown C2C12 myocytes (*N* = 4–5). Molecular and metabolic assays and proliferation experiments were performed using stable Nr2f6 knockdown and Nr2f6 overexpression C2C12 cell lines (*N* = 3–6). Nr2f6 content was evaluated in lipid overload models *in vitro* and *in vivo* (*N* = 3–6). *In vivo* experiments included Nr2f6 overexpression in mouse *tibialis anterior* muscle, followed by gene array transcriptomics and molecular assays (*N* = 4), *ex vivo* contractility experiments (*N* = 5), and histological analysis (*N* = 7). The conservation of Nr2f6 depletion effects was confirmed in primary skeletal muscle cells of humans and mice.

**Results:**

Nr2f6 knockdown upregulated genes associated with muscle differentiation, metabolism, and contraction, while cell cycle‐related genes were downregulated. In human skeletal muscle cells, Nr2f6 knockdown significantly increased the expression of myosin heavy chain genes (two‐fold to three‐fold) and siRNA‐mediated depletion of Nr2f6 increased maximal C2C12 myocyte's lipid oxidative capacity by 75% and protected against lipid‐induced cell death. Nr2f6 content decreased by 40% in lipid‐overloaded myotubes and by 50% in the skeletal muscle of mice fed a high‐fat diet. Nr2f6 overexpression in mice resulted in an atrophic and hypoplastic state, characterized by a significant reduction in muscle mass (15%) and myofibre content (18%), followed by an impairment (50%) in force production. These functional phenotypes were accompanied by the establishment of an inflammation‐like molecular signature and a decrease in the expression of genes involved in muscle contractility and oxidative metabolism, which was associated with the repression of the uncoupling protein 3 (20%) and PGC‐1α (30%) promoters activity following Nr2f6 overexpression *in vitro*. Additionally, Nr2f6 regulated core components of the cell division machinery, effectively decoupling muscle cell proliferation from differentiation.

**Conclusions:**

Our findings reveal a novel role for Nr2f6 as a molecular transducer that plays a crucial role in maintaining the balance between skeletal muscle contractile function and oxidative capacity. These results have significant implications for the development of potential therapeutic strategies for metabolic diseases and myopathies.

## Introduction

Muscle contraction is a highly coordinated process initiated by the transmission of the action potential from efferent neurons to the muscle. Subsequent depolarization of the muscle fibre propagates along the sarcolemma, stimulating the release of calcium ions from the sarcoplasmic reticulum, and promoting myosin‐actin cross‐bridge cycling and force production. An accessory metabolic machinery that generates energy is necessary to support these processes, and disruption in muscle structure or metabolism leads to pathologies, such as Duchenne's syndrome,^1^ sarcopenia,^2^ and cachexia.^3^ Dynamic crosstalk between energetic status, muscle development, mechanical stress, and transcriptional changes is crucial to maintaining muscle function. In this context, the nuclear receptor family of transcription factors (NRs) is of particular interest as they are potentially regulated by small molecules, such as metabolites and hormones.^4^

Although the transcriptional landscape for metabolic‐functional signalling has been extensively studied in pathological and physiological conditions, a broader role of some NRs has only recently been recognized and the role of many members remains vague.^5^ NRs have a modular architecture displaying a ligand‐binding domain (LBD) and a zinc‐finger DNA binding domain (DBD) and can be further grouped in endogenous, orphan, or adopted NRs according to the presence of an endogenous ligand, the absence of a known ligand or if a new ligand was recently identified, respectively.^6^ The classical mechanistic model proposes that a small molecule binds to the LBD, changing its conformation to one of higher affinity for a transcriptional co‐regulator, such as the PPARγ coactivator‐1 alpha (PGC‐1α), which can mediate transcriptional modulation through the recruitment of chromatin modifiers and basal transcriptional apparatus.^7^ The orphan nuclear receptor Nr2f6, also named Ear2, has been characterized in several tissues and organs, such as adipose, thyroid, liver, brain, and the immune system, where it plays different and even antagonistic roles.^8^ Nr2f6 can impair adipocyte differentiation, increase cancer cell proliferation, and promote the development of fatty liver disease by upregulating CD36.^9^ So far, the most extensively defined role of Nr2f6 is in the immune system, in which it directly and strongly suppresses interleukins 17, 21, 2, and interferon γ transcription by interacting with the NFAT/AP‐1 complex at the promoters of these genes.^10^, ^11^

Nr2f6 has been reported both as a transcriptional repressor and activator, but the context that defines its activity state is unknown. Recently, Nr2f6 was classified as a stripe transcription factor,^12^ that is, it can bind to low‐complexity motifs together with other transcription factors in a broad range of promoters, regulating chromatin accessibility. This suggests that Nr2f6's function in different environments depends on its DNA occupancy and interaction with other transcription factors. Therefore, it is uncertain if the current understanding of Nr2f6's role can be extended to other tissues like skeletal muscle. Hence, we sought to characterize, for the first time, the molecular mechanisms, and functional roles of Nr2f6 in skeletal muscle biology both *in vitro* and *in vivo*. *In vitro* approaches identified Nr2f6 as a repressor of PGC‐1α and uncoupling protein 3 (UCP3) gene expression, consistent with the improvement in cells' resilience to lipid toxicity and enhanced oxidative capacity in Nr2f6 loss‐of‐function models. Nr2f6 gain‐of‐function *in vivo* promoted an inflammatory transcriptional signature, and upregulation of core genes of cell cycle progression, culminating in loss of muscle mass, changes in fibre type, and impairment of force production.

## Methods

The main reagents, tools, and models necessary for replicating the reported results are listed in Supporting Information S3. Details of all methods are described in the Methods section of the Supporting information S1.

### Cell culture

Human primary skeletal muscle cells were isolated from healthy donors,^13^ 55 ± 5 years old, 25.6 ± 1.5 kg m^−2^ BMI. After confluence, growth media was switched to fusion media, and cells were cultivated for another 9 days. C2C12, MEF, and HEK cells were maintained in DMEM high glucose with supplements. Bovine serum was switched to horse serum to induce myogenesis in C2C12, and experiments were performed 5 days later.

### Primary mouse skeletal muscle cells

Myoblasts were isolated as described^14^ and maintained for 2 days in DMEM high glucose with supplements. Myogenesis was induced for 5 days by removing foetal bovine serum when confluence was reached.

### Animals

Male C57Bl6/J mice were maintained at 12/12 h light/dark cycle under controlled temperature and humidity, and *ad libitum* access to food and water. For high‐fat diet experiments, 4‐week‐old C57Bl6/JUnib mice were fed for 16 weeks.

### Reactive oxygen species measurement

Cells were incubated with 5 nM MitoSOX or 5 μM dihydroethidium and fluorescence intensity was measured in a plate reader. Assays were normalized using Crystal Violet.

### Reverse transcription‐quantitative PCR

Total RNA was extracted with TRIzol and cDNA was synthesized with a high‐capacity reverse transcription kit. Primers and probes are listed in Supporting Information S2. Expression is displayed as fold‐change over the indicated control.^15^, ^16^

### RNA sequencing

Library was constructed with TruSeq Illumina Total RNA Stranded and a HiSeq X used for sequencing. Data was processed using Trimmomatic,^17^ RNA Star, featureCounts, and EdgeR. Pathway enrichment analysis was done using g:Profiler and interaction networks were analysed with CytoScape.

### Fatty acid treatment

Palmitate (500 μM) and oleate (500 μM) were conjugated with 1% fatty acid‐free bovine serum albumin in cell media and cells were treated for 20 h.

### Promoter transactivation assays

MEF cells were transfected with UCP3 7 kbp^18^ or PGC‐1α 2 kb promoters, Renilla luciferase, and either empty vector or Nr2f6 coding plasmid. Luciferase activity was measured with the DualGlo Luciferase Reporter kit. In knockdown assays, cells were transfected with siRNAs 1 day before the transfection with reporter plasmids.

### siRNA knockdown

C2C12 were transfected with 200 nM non‐target siRNA or siNr2f6 concomitantly with the differentiation media switch and experiments were performed 3 days later. Primary human skeletal muscle cells were transfected twice with 5 nM siScr or siNr2f6 and the experiments were performed on fully differentiated myotubes.

### Stable cell lines

Retroviral particles were used to generate stable shNr2f6 (TRCN0000026147) and Nr2f6 overexpressing C2C12 cells.

### Electroporation

Mice *tibialis anterior* were injected with empty or Nr2f6‐myc‐flag plasmids and voltage was applied. Experiments were performed 9 days after electroporation on 13‐week‐old mice.

### *Ex vivo* contraction

Mouse *flexor digitorum brevis* were electroporated and dissected 8 days later. Measurements were conducted at optimal muscle length and maximal force was calculated as the difference between peak force and baseline. Time to fatigue was measured as the time to reach 50% peak intensity.

### Myosing heavy chain staining

*Tibialis anterior* were dissected and immediately frozen in nitrogen‐cooled isopentane. Muscle slices were blocked and probed with primary antibodies. Whole sections were imaged in a fluorescent scanning microscope at 20× magnification.

### Oxygen consumption assays

Oxygen consumption rates (OCR) were measured in a Seahorse XF24 extracellular flux analyser.

### Lactate measurement

Lactate was quantified by the reverse reaction of l‐lactate dehydrogenase and normalized using Crystal Violet.

### Western blot

Protein was extracted with RIPA buffer and loaded into gradient SDS‐PAGE gels. Separated proteins were transferred to PVDF membranes and detected with ECL. Data are shown as fold‐change over control.

### Microarray

Gene expression in *tibialis anterior* was assessed with an Affymetrix Whole Transcript (WT) Assay kit probed in a CGAS cartridge for Clariom S (mouse).

### Cell death assays

Cell‐death assays were performed as described,^19^ and fluorescence intensity was measured in a plate reader.

### Cell doubling time

Cells were plated and counted in a Neubauer chamber every 24 h.

### ATP measurement

CellTiter‐Glo Luminescent Cell Viability Assay kit was used for measuring absolute ATP concentrations.

### Bioinformatic analysis of public datasets

Nr2f6 ChIP‐seq data from HepG2 and K562 cells (GSM2797593 and GSM2534343^20^, ^21^) and C2C12 RNA‐seq (GSE4694)^22^ were used. Pathway enrichment was performed using g.profiler, and Nr2f6 response elements were identified using RSAT.

### Statistical analysis and quantification

GraphPad Prism v7.0 was used for plotting and statistical analysis. Data normality was confirmed by the Shapiro–Wilk test.

## Results

### Nr2f6 regulates the transcriptional landscape of myogenesis and metabolism in skeletal muscle cells

**Figure 1:**
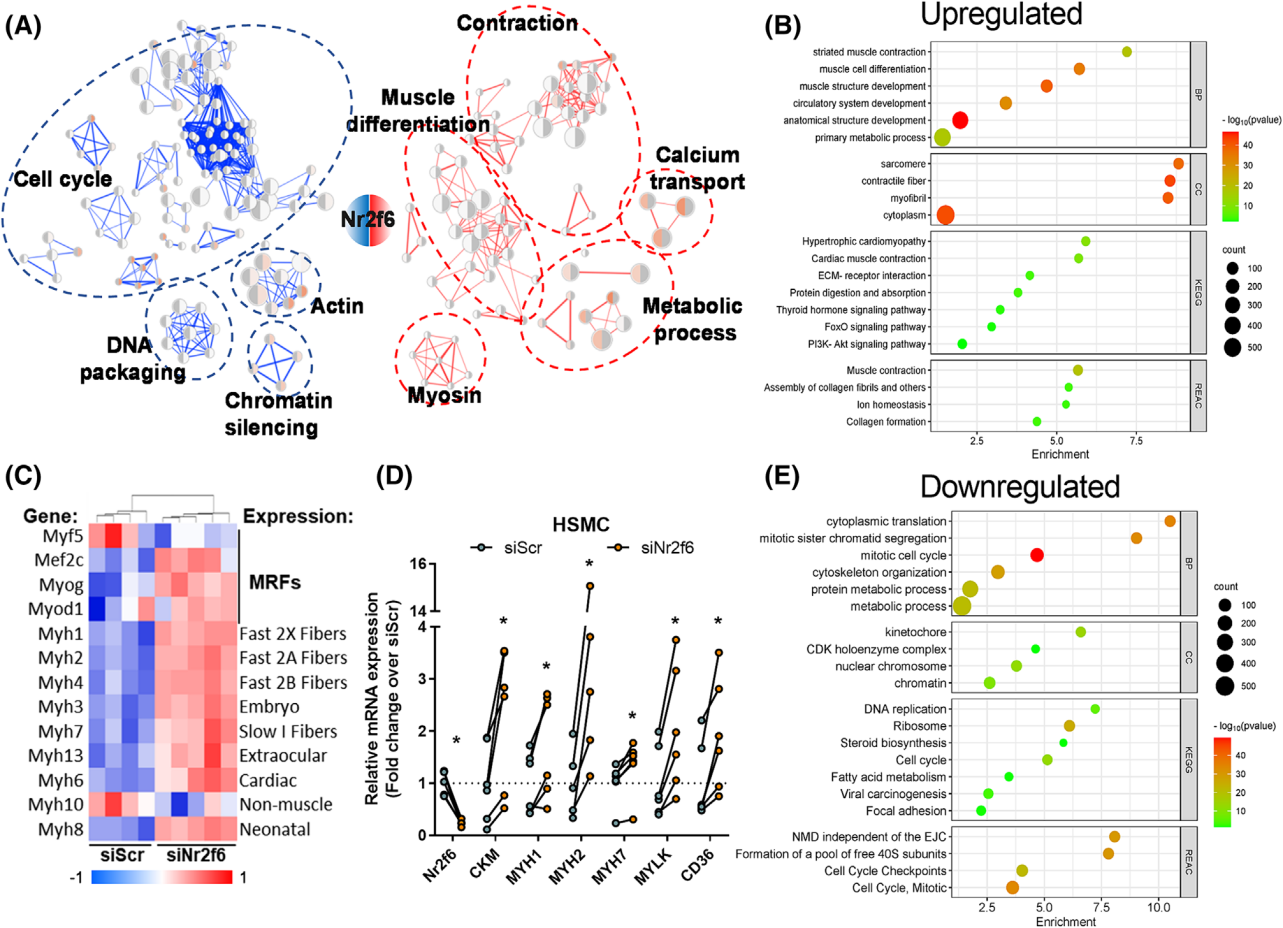
Nr2f6 knockdown derepresses the expression of genes involved in metabolism and myogenesis. (A) Network of ontology terms enriched in the differentially expressed genes in the transcriptomics of transient Nr2f6 knockdown in C2C12 cells. Groups of similar terms were manually curated and encircled as indicated. Upregulated elements in red and downregulated in blue (*N* = 4–5). (B, E) Gene ontology enrichment of downregulated and upregulated genes. (C) Panel of myogenic differentiation markers differentially regulated by Nr2f6 knockdown with myogenic regulatory factors (MRFs) and myosin isoforms and their respective fibre expression patterns.^S69,S70^ (D) Gene expression measured by RT‐qPCR of markers of myogenic differentiation in primary human skeletal muscle myotubes (HSMC) transfected with control non‐target RNAi (siScr) or siNr2f6 (*N* = 5–6). Circles represent individual donors. *P < 0.05 using ratio paired two‐tailed Student's *t*‐test.

Whole‐body and *in vitro* genetic manipulations of Nr2f6 have been conducted,^9^, ^23^, ^24^, ^25^ but its role in the skeletal muscle is underexplored. We depleted Nr2f6 (75%) in C2C12 myocytes using siRNA and verified the effects on the transcriptome landscape through RNA‐seq (Figure S1A). The 1849 differentially expressed genes, 920 upregulated and 939 downregulated, could be grouped into five main classes, with increased expression of genes related to muscle differentiation, contraction, and metabolism and decreased expression of genes with roles in mitosis and DNA packaging (Figure 1A,B,E). Among the 20 most significant genes, 11 are linked to muscle contraction (*RYR1*, *TTN*, *MYH3*, *MYH2*, *ACNT2*, *ATP2A1*, *MYL1*, *TNNC2*, *MYOM3*, *CACNA1S*, and *LRP4*) and other three compose the cytoskeleton (NEB, MACF1, and XIRP1), all upregulated by Nr2f6 knockdown (Figure S1B). Accordingly, canonical markers of myogenesis including muscle regulatory factors (MRFs) and myosins (Figure 1C) were broadly upregulated. Ontologies of the upregulated genes were enriched with sarcomere, contractile fibre, and cytoplasm location terms. Our transcriptome directly correlated with a public C2C12 differentiation dataset (Figure S1C), indicating an association between Nr2f6 levels and myogenic potential. Myogenesis demands withdrawal of the cell cycle and both processes are concertedly coordinated^26^, ^27^ and downregulated genes were related to cell division, with enrichment DNA replication, packaging, and chromosome segregation genes, including essential components of the mitosis progression, such as *CDC25B/C* phosphatases and *CDK1/4* kinases, that induce quiescence and halt cycle in muscle progenitors when down‐regulated.^28^, ^29^, ^30^ Considering the differences between mouse and human myogenic transcriptional landscape^31^ we verified whether Nr2f6 depletion effects are conserved in primary human skeletal muscle cells. Consistently, the expression of *MYH1/2/7*, muscle creatine kinase, and myosin light chain kinase 1 were upregulated by Nr2f6 knockdown in human myotubes (Figure 1D). Because increased cellular oxidative capacity and the activation of the PI3K pathway are required for the completion of myogenesis,^32^, ^33^ we investigated whether the genes of major pathways of glycolysis and β‐oxidation were also affected by Nr2f6 depletion and found that several energy sensors such as *AKT2*, *PRKAG3* subunit of AMP‐activated protein kinase (AMPK) and the mTOR complex were upregulated (Figure S1D). Altogether, the changes in the myocyte's global transcriptome indicate that Nr2f6 represses key gene networks associated with myoblast differentiation and metabolism.

### Nr2f6 depletion improves oxidative metabolism and enhances lipid handling capacity

**Figure 2:**
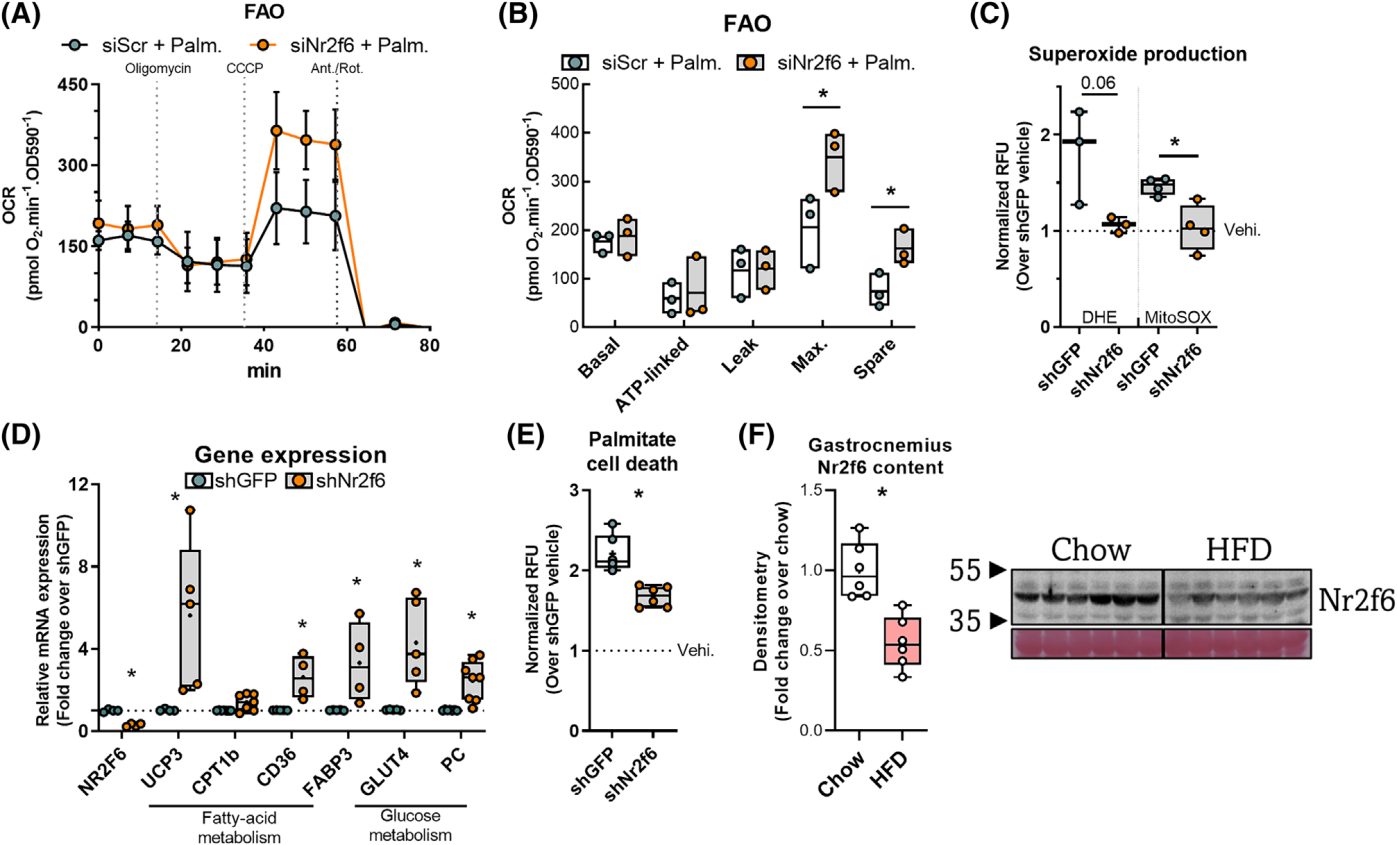
Nr2f6 depletion increases fatty acid oxidation and protects cells against lipid‐induced stress. (A) Fatty acid‐dependent oxygen consumption (FAO) assay in control siScr and siNr2f6 C2C12 myocytes using palmitate (palm.) as substrate. Data displayed as mean ± SD. (B) Calculated respiratory parameters of the FAO assay are displayed as a line on the mean and minimum to max bars (*N* = 3). *P < 0.05 using unpaired two‐tailed Student's *t*‐test. (C) Mitochondrial and total superoxide production following palmitate treatment in shGFP and shNr2f6 stable C2C12 cells (*N* = 3–4). (D) Relative gene expression using RT‐qPCR in stable Nr2f6 knockdown (shNr2f6) C2C12 cells and control shGFP stable cells (*N* = 4–8). (E) Control shGFP or Nr2f6 knockdown cells were exposed to 500 μM palmitate (palm.), or vehicle (vehi.) for 20 h, and relative cell death was measured by propidium iodide staining (*N* = 5). (F) Relative Nr2f6 protein content in the gastrocnemius of mice fed with a control chow diet or high‐fat diet (HFD) for 16 weeks. Inlet: Representative western blot image (*N* = 6). Boxplot with whiskers spanning minimum to maximal and box edges 25th–75th percentile, the line at the median and + at the mean. *P < 0.05 using unpaired two‐tailed Student's *t*‐test.

Given the enrichment of genes related to energetic metabolism, we examined the functional effects of Nr2f6 knockdown on oxidative capacity. Maximal respiration and spare capacity were increased in oxygen consumption (Figure 2A,B) using palmitate as the major substrate. In high‐glucose media, there was no difference between control and knockdown (Figure S2A, B). However, knockdown cells showed reduced extracellular acidification rates and lower extracellular lactate concentration, without decreasing ATP concentrations (Figure S2C, D, E). We hypothesized that these cells would be protected against lipid overload, and generated stable Nr2f6 knockdown (70%) C2C12 cell line to study the effects of sustained Nr2f6 depletion (Figure 2D). Nr2f6 knockdown attenuated palmitate‐induced cell death (Figure 2E) and prevented the palmitate‐induced increase in mitochondrial and cytosolic superoxide (Figure 2C). Accordingly, Nr2f6 knockdown increased the expression of the glucose transporter *GLUT4*, the anaplerotic enzyme pyruvate carboxylase (PC), and fatty acid transporters (Figure 2D). Next, we verified whether Nr2f6 is modulated by palmitate treatment *in vitro* and by high‐fat diet in mice, as models of increased lipid oxidation and supply.^34^ These conditions reduced Nr2f6 expression, indicating a role as an energy stress response gene that facilitates metabolic adaptations to improve lipid oxidation (Figures 2F and S2F‐I). To characterize the Nr2f6 cistrome, we explored ENCODE Nr2f6 chromatin immunoprecipitation‐sequencing (ChIP‐seq) data from two contrasting cell lines, human hepatocarcinoma (HepG2) and human acute myeloma (K562). There were 5610 common Nr2f6 binding sites, corresponding to 1868 unique genes with peaks within the promoter. Gene ontology (Figure S2J) indicated enrichment in metabolic processes, respiration, stress response, organelle organization, and lipid metabolism terms. Consistently, there was a significant enrichment of kinases of the insulin signalling pathway in the Nr2f6 knockdown transcriptome (Figure S2K). Collectively, the data provide evidence that Nr2f6 inhibition protects against lipid overload by increasing lipid handling capacity, possibly by a concerted upregulation of mitochondrial and cytosolic lipid transporters, mitochondrial proteins, and TCA cycle anaplerotic genes.

### Transrepression of PGC‐1α and UCP3 transcription by Nr2f6

**Figure 3:**
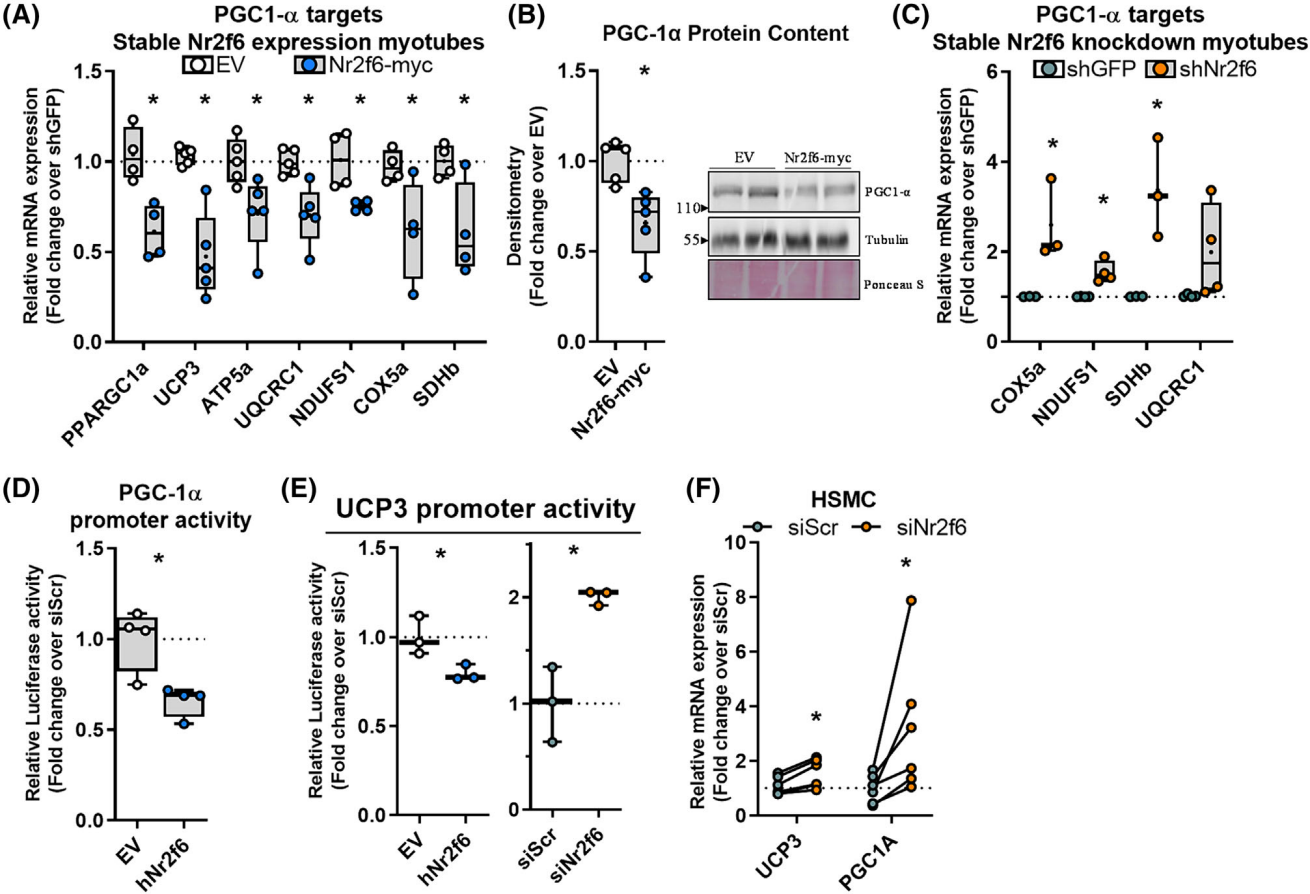
Nr2f6 inhibits PGC1‐α and UCP3 gene expression. (A) Relative gene expression using RT‐qPCR in stable Nr2f6‐myc overexpression myotubes (*N* = 4–5). (B) Densitometry and representative images of PGC‐1α western blot in stable Nr2f6‐myc overexpression myotubes (*N* = 5). (C) Relative gene expression by RT‐qPCR in stable Nr2f6 knockdown myotubes (*N* = 3–5). (D) PGC‐1α 2kbp luciferase reporter assay in HEK293 cells overexpressing HA‐tagged Nr2f6 (Nr2f6‐HA) or control empty vector (EV). (E) Luciferase activity of UCP3 promoter transactivation assay in cells transfected with Nr2f6‐HA or siNr2f6 (*N* = 3). (F) Relative gene expression by RT‐qPCR in human primary skeletal myotubes (HSMC) transfected with siNr2f6 or siScr (*N* = 6). Boxplot with whiskers spanning minimum to maximal and box edges 25th–75th percentile, the line at the median and + at the mean. *P < 0.05 using ratio paired two‐tailed Student's *t*‐test for the experiments with human cells and unpaired for the other comparisons.

We recently provided evidence that PGC‐1α regulation of *UCP3* transcription is essential for maintaining myotube viability during lipid overload.^35^ Considering a similar phenotype in Nr2f6 knockdown, we investigated whether Nr2f6 regulates the same pathway. Nr2f6 overexpression (Figure S3C, D) downregulated both *UCP3* and *PGC‐1α* in myotubes, also decreasing PGC‐1α protein content and its mitochondrial electron transfer chain (ETC) target genes (Figure 3A,B). Conversely, Nr2f6 knockdown increased PGC‐1α ETC targets (Figure 3C). PPARGC1A and UCP3 promoters were inhibited by Nr2f6 overexpression, and the latter was increased by Nr2f6 knockdown (Figure 3D,E). Nr2f6 binding motifs were found within both UCP3 and PPARGC1A promoters (Figure S3E, F) and the match in the UCP3 promoter overlapped an open chromatin region and anchoring sites of Myod1 and Myogenin known regulators of UCP3 transcription. Importantly, the inhibitory effects of Nr2f6 on *UCP3* and *PGC‐1α* expression were conserved in primary myotubes of both humans (Figure 3F) and mice (Figure S3A). UCP3 transcription is regulated by peroxisome proliferator‐activated receptors (PPARs) and oestrogen‐related receptors (ERRs) in skeletal muscle^36^, ^37^; however, transactivation of their response elements remained unchanged in Nr2f6 knockdown (Figure S3B). Given that UCP3 is a PGC‐1α target, these results suggest that Nr2f6 can regulate UCP3 expression indirectly, via PGC‐1α transcriptional regulation, or by direct modulation of UCP3 promoter activity.

### Nr2f6 promotes cell proliferation and represses genes implicated in muscle contraction and oxidative metabolism

**Figure 4:**
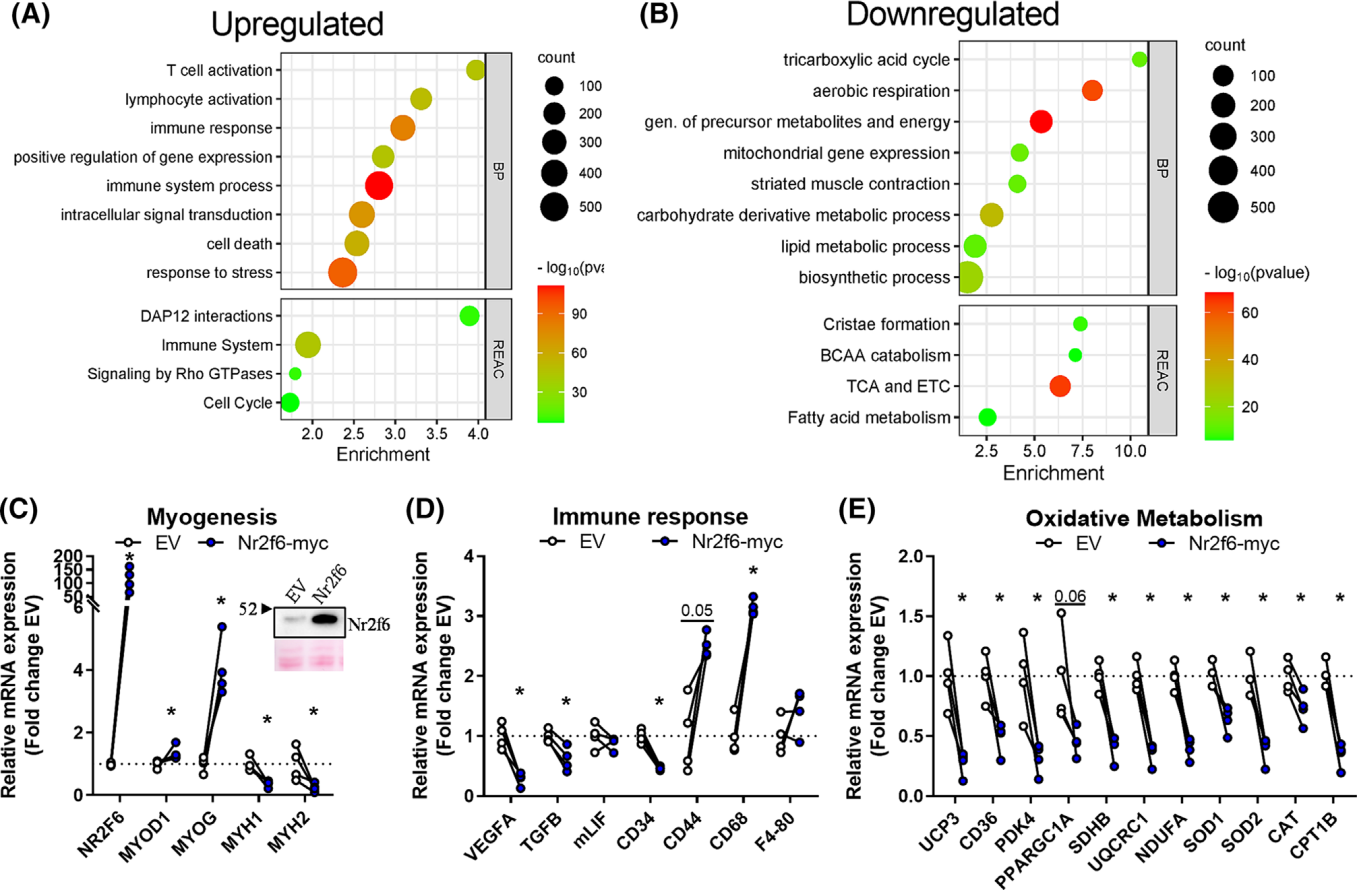
The transcriptional landscape of Nr2f6 overexpression exhibits a decrease in metabolism and an increase in inflammatory markers (A, B) Gene ontology enrichment of downregulated and upregulated genes. (C, D, E) Validation of selected markers modulated in the microarray by RT‐qPCR (*N* = 4). Insert on (C): Representative western blot for validation of Nr2f6 overexpression in the *tibialis anterior* samples in empty vector control (EV) and Nr2f6 electroporated (Nr2f6‐myc) muscles. Circles represent individual samples. *P < 0.05 using ratio paired two‐tailed Student's *t*‐test. The numbers above some bars indicate the *P*‐value. CAT, catalase; CD, cluster differentiation; CPT1B, carnitine palmitoyltransferase 1B; F4‐80, EGF‐like module‐containing mucin‐like hormone receptor‐like 1; mLIF, monocyte locomotion inhibitory factor; MYH1 and MYH2, myosin heavy chain 1–2; MYOD1, myogenic differentiation 1; MYOG, myogenin; NDUFA1, NADH:Ubiquinone oxidoreductase subunit A1; PDK4, pyruvate dehydrogenase kinase 4; PPARGC1A, PPARG coactivator‐1 α; SDHB, succinate dehydrogenase b; SOD1 and SOD2, superoxidase dismutase 1–2; TGFB, transforming growth factor beta; UCP3, uncoupling protein 3; UQCRC1, ubiquinol‐cytochrome c reductase core protein 1; VEGF, vascular endothelial growth factor.

We explored the effects of Nr2f6 overexpression *in vivo* by electroporation of mice *tibialis anterior* muscle. Microarray transcriptomics revealed 3796 (FDR < 0.05, |fold change| > 2) differentially expressed genes, 1915 downregulated and 1781 upregulated, with Nr2f6 overexpression having a major effect on the hierarchical clustering (Figure S4A). Consistent with reports that highlight Nr2f6 as a gatekeeper of the immune response,^24^ there was an enrichment of ontologies of processes and pathways associated with the immune system (Figure 4A). RT‐qPCR validation indicated that the markers of lymphocyte activation, *CD44*, macrophage/monocyte activation, *CD68*, and macrophages, *F4–80* , were upregulated by Nr2f6 overexpression (Figure 4D). *TGFb*, a potent inhibitor of haematopoietic cell activation, and the marker for endothelial and non‐differentiated haematopoietic cells, VEGFa, were downregulated. These results suggest that Nr2f6 might modulate immune resident cells and/or promote the invasion of circulating cells. Downregulated genes were enriched in energetic metabolism, mitochondria, and muscle contraction terms (Figure 4B), consistent with the functional phenotypes described *in vitro*. Nr2f6 overexpression also increased the expression of Myogenin and *MYOD*, however, their downstream targets myosin heavy chains 1 and 2 were decreased (Figure 4C).

*UCP3* and *PGC‐1α* were also repressed by Nr2f6 *in vivo* (Figure 4E). The lipid transporters CD36 and CPT1B, and subunits of the ETC, upregulated by Nr2f6 knockdown *in vitro*, were downregulated by Nr2f6 overexpression. Additionally, the expression of reactive oxygen species scavengers *SOD1*, *SOD2*, and catalase genes was decreased (Figure 4E). Collectively, these findings further confirm our functional results *in vitro* and indicate that mitochondrial activity is impaired by Nr2f6 overexpression.

### Overexpression of Nr2f6 exacerbates muscle mass wasting and impairs force production

**Figure 5:**
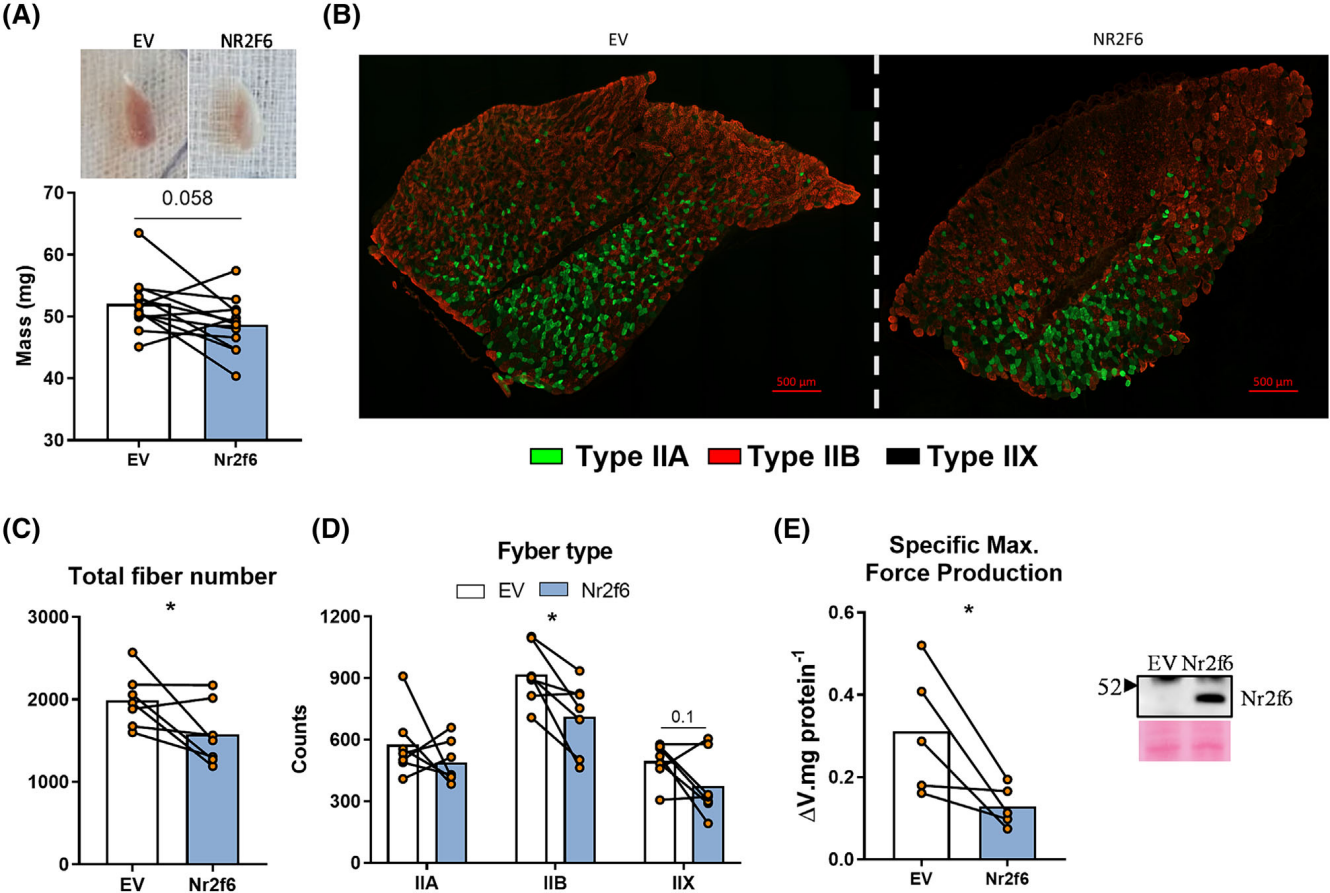
Overexpression of Nr2f6 induces muscle atrophy and impairs muscle force production. (A) Weight of *tibialis anterior* muscles (TA) electroporated with empty vector (EV) or Nr2f6‐myc coding plasmid. Top: Representative photo of electroporated muscles (*N* = 12). (B) Representative images of myosin heavy chain staining in the electroporated TAs for fibre type determination. In green, MHC IIA; in red, MHC IIB; unstained fibres as IIX. No substantial number of MHCI fibres were stained, therefore the corresponding channel was omitted (*N* = 7). (C, D) Total and type‐segmented fibre counts (*N* = 7). (E) *Ex vivo* contraction maximal force production in FDB muscles electroporated with control empty vector (EV) or Nr2f6‐coding plasmid (*N* = 5). Data are displayed as individual animals and bars at the mean. *P < 0.05 using ratio paired two‐tailed Student's *t*‐test.

As Nr2f6 negatively affects muscle contraction and development genes, we considered whether Nr2f6 gain‐of‐function would impair muscle morphology and function. Nr2f6 overexpressing *tibialis anterior* (TA) muscles weighted less and were visually paler (Figure 5A). Nr2f6 overexpression reduced (20%) the number of myofibres, mainly due to the decrease (25%) in type IIB fibres (Figure 5B–D).

Together with the increase in cell death‐related genes (Figure S4B) and the increase in the atrogenes cathepsin and calpain 2^38^ (Figure S4B), this characterizes a state of atrophy and hypoplasia. The functional incurrences of these molecular disturbances were investigated by *ex vivo* contractions *of Nr2f6‐overexpressing flexor digitorum brevis* (FDB). Considering the length constraints of these experiments, FDB was a suitable model, due to its short size, ease of access for electroporation, and similarity with TA fibre composition.^39^ Mass‐corrected maximal force production was considerably reduced (60%) by Nr2f6 overexpression (Figure 5E). No effects were observed in the time to fatigue between control and treated groups (Figure S4C). As neuronal signals are bypassed by direct electric stimulation, effects on the transmission of the action potential are disregarded and fatigability is mostly induced by detriments in Ca^2+^ cycling^40^; therefore, we cannot exclude the possibility that fatigability is affected by Nr2f6 *in vivo*. Altogether, these findings strongly suggest that Nr2f6 induces muscle loss, worsened by an overactivation of the immune system and an imbalance between satellite cell proliferation and differentiation. Nr2f6 expression showed a strong tendency (*P* = 0.056) towards an increase in *gastrocnemius* of elder mice (S5B), which displayed upregulated Myod1 and myogenin.^S1,S2^ Mining public datasets (Figure S5A), we found that Nr2f6 is increased in biopsies from hereditary spastic paraplegia (HSP)^S3^ and decreased in dermatomyositis (DM). HSP causes severe muscle atrophy, accompanied by energetic dysfunction.^S4^ DM muscles are weaker^S5,S6^ and are characterized by bundles of atrophic and regenerating muscle fibres,^S7^ suggesting that Nr2f6 may have divergent expression within the same tissue despite overall downregulation. These findings imply a two‐way association between Nr2f6 content and muscle function in various physiological and pathophysiological conditions.

### Myoblast proliferation rates are governed by Nr2f6

**Figure 6:**
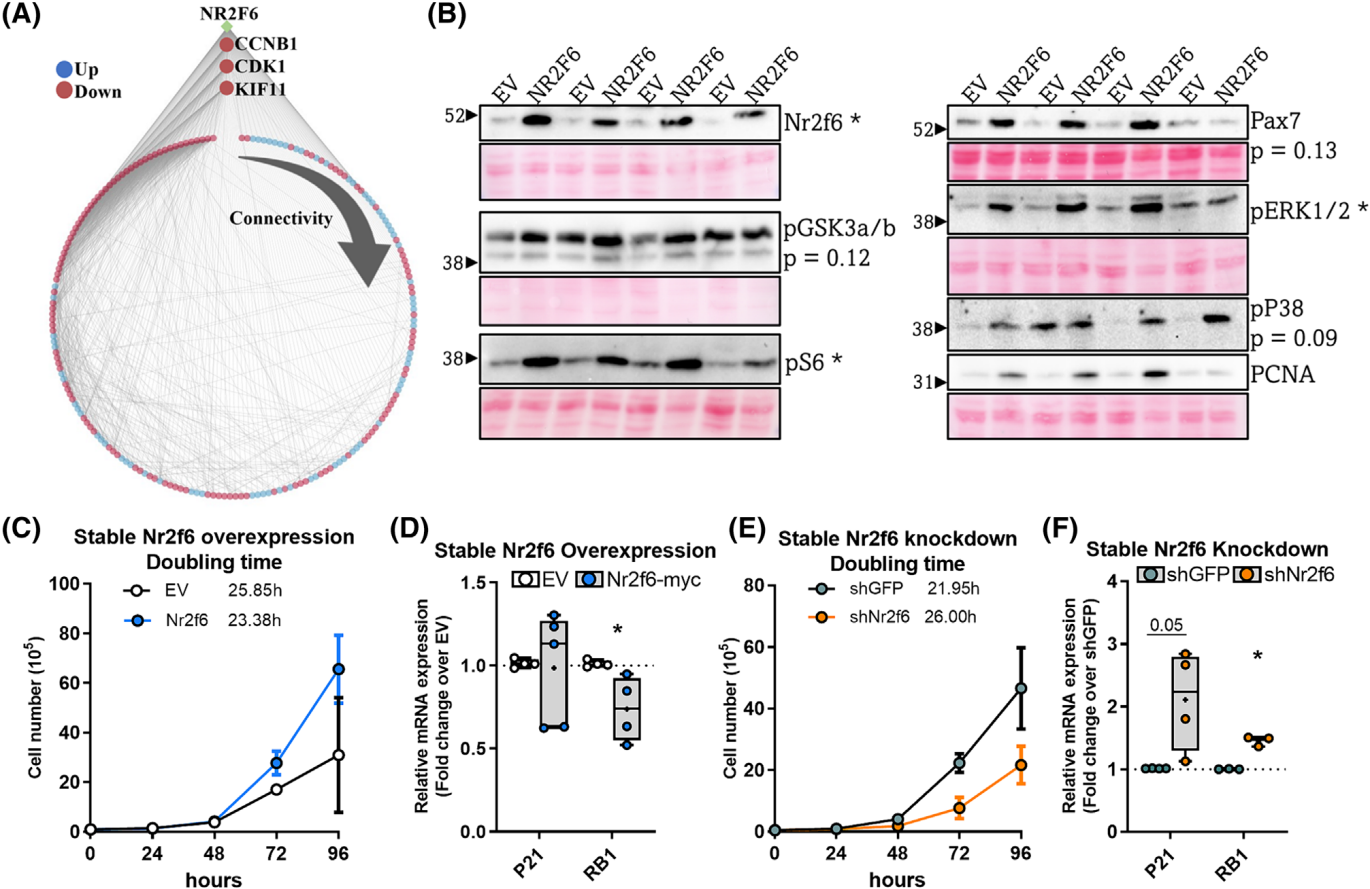
Nr2f6 increases myoblast proliferation rates. (A) Interaction network of genes consistently regulated by Nr2f6 overexpression and knockdown and with detected Nr2f6 binding motif at the promoter region. In blue: Genes downregulated; in red: Genes upregulated. The number of connections of each gene increases clockwise. (B) Representative images of the western blot of control (EV) or Nr2f6‐myc electroporated *tibialis anterior* muscles. Densitometric quantitation of the indicated protein bands is provided in Figure S5B (*N* = 4). (C, E) Proliferation curves of stable C2C12 cell lines and the calculated doubling time. (D, F) RT‐qPCR of cell cycle arrest markers in Nr2f6 knockdown and overexpression stable cell lines, respectively (*N* = 4–6). Boxplot with whiskers spanning minimum to maximal and box edges 25th–75th percentile, the line at the median and + at the mean. *P < 0.05 using unpaired Student's *t*‐test. The numbers above some bars and next to antibodies indicate the *P*‐value when >0.05.

Comparing the transcriptomes of Nr2f6 overexpression in TAs and Nr2f6 depletion in myocytes, we found 706 differentially expressed genes (DEGs) in common, whereby 446 genes were modulated in opposite directions (Figure S6A). Scanning these gene's promoters with Nr2f6 binding motifs available on JASPAR, matched 206 unique genes, whereby 73 were upregulated and 133 downregulated in the Nr2f6 overexpression microarray. Examination of the interaction network of these high‐confidence targets had the most connected genes associated with the cell cycle and upregulated by Nr2f6 overexpression (Figure 6A), which emphasizes the role of Nr2f6 as a promoter of cell division and reinforces the dysplastic phenotype observed in the gain‐of‐function experiments *in vivo*. Analysis of ENCODE Nr2f6 ChIP‐seq data (Figure S7A), provided additional insights into the Nr2f6‐driven regulatory networks and addressed possible regulatory partners. *De novo* motif enrichment analysis was performed in Nr2f6 peaks coincident in both cell types and the most probable transcription factors were attributed to the enriched motifs. As expected, MEME‐ChIP shows the highest enrichment of the Nr2f6 binding sequence along with other NRs (Figure S7D,E), although their binding matrices are similar, NRs with key functions in muscle metabolism and differentiation and that interact with Nr2f6, such as RXRA,^S24^ NR2F2,^S24,S25^ and NR2C1^S25^ were also considerably enriched. Outside the NR family, GATA binding protein 5 (GATA5), HNF1 homeobox A (HNF1A), ETS domain‐containing protein (ELK1), and FBJ osteosarcoma oncogene (FOS) response elements were also frequent and followed Nr2f6's motif spatial distribution, suggesting a potential interaction at gene promoters to regulate transcription.

To find potential direct targets, we extracted common peaks in both ENCODE Nr2f6 ChIP‐seq experiments and crossed them with our Nr2f6 knockdown transcriptome (Figure S7A), resulting in 231 unique genes significantly modulated by Nr2f6 knockdown, 3 modulated over two‐fold change (Figure S7B): Insulin‐like growth factor 2 mRNA‐binding protein 1 (IGF2BP1), adhesion G protein‐coupled receptor F3 (ADGRF3), and augurin precursor (ECRG4). IGF2BP1 is an RNA‐binding protein with important functions in organ development and proliferation of cancer cells and myoblasts.^S8,S9,S10^ ECRG4 is a secreted peptide regarded as a tumour suppressor^S11^ and is required for proper cardiomyocyte differentiation.^S12^ These phenotypes are consistent with the presented Nr2f6 knockdown models, and further studies should investigate the functional relationship between Nr2f6 and ECRG4. The scientific literature is scarce regarding ADGFR3, although it has been associated with neuroendocrine tumors^S13^. Accordingly, the protein content of several markers of cell proliferation and stemness was increased in Nr2f6 overexpressing TAs (Figures 6B and S6B), including the proliferating cell nuclear antigen (PCNA), a fundamental marker for cell proliferation^S14^; Pax7, muscle‐specific satellite cell marker; the phosphorylation of the stemness marker GSK3a/b,^S15,S16^ and the proliferation markers ERK1/2, p38,^S17,S18^ and S6.^S19,S20^ These modulatory effects were consistent across animals, with a single mouse displaying deviant behaviour; in these cases, comparisons with *P*‐value <0.15 are shown and were considered relevant to explain the observations. In cancer cells, Nr2f6 overexpression and knockdown can promote or inhibit cell proliferation, respectively.^S21–S23^ Doubling time experiments confirm that this effect is also conserved in C2C12 myoblasts, with an increase (4 h) in the average doubling time by Nr2f6 depletion and a decrease (3.5 h) by Nr2f6 overexpression (Figure 6C,E). *RB1* and *P21*, major markers of cell cycle inhibition, are upregulated by Nr2f6 knockdown and *RB1* is downregulated by Nr2f6 overexpression (Figure 6D,F). Collectively, these results demonstrate that Nr2f6 works as a major promoter of cell cycle progression in the skeletal muscle, possibly by direct modulation of transcriptional networks implicated in cell division.

## Discussion

Numerous nuclear receptors are necessary for the maintenance of muscle mass.^S26, S27, S28^ For example, whole‐body knockout of Nr1d1 (Rev‐ERBα and Ear‐1) increases atrophic genes and low‐diameter fibres and decreases muscle mass.^S29^ More broadly, nuclear receptor co‐repressor 1 (NCoR1) muscle‐specific knockout induces hypertrophy and increases oxidative metabolism.^S30^ Here, using several skeletal muscle models we gathered evidence that Nr2f6 overexpression disrupts myogenesis *in vivo* and *in vitro*, and activates myoblast proliferation. Remarkably, Nr2f2 (COUP‐TFII), an Nr2f6 interactor, is among the few nuclear receptors known to promote muscle wasting.^S31^ Nonetheless, Nr2f2 expression in myogenic progenitors impairs muscle differentiation by directly repressing genes associated with myoblast fusion and proliferation,^S32^ implying that these NRs regulate distinct phases of myogenesis. Future studies should address the redundancy of these NRs in muscle function.

Most of the genetically modified models suggest that NRs are involved in a general activation of oxidative metabolism.^S26^ For example, Nr4a3 muscle‐specific transgenic mice display enhanced mitochondrial density and fast‐to‐slow fibre switch.^S33^ Mice lacking NRs coactivators, such as PGC‐1α and MED1,^S34^ or overexpressing the NRs co‐repressor RIP140,^S35^ show decreased mitochondrial density and fewer oxidative fibres. We demonstrate that Nr2f6 is an exception to this model as it reduces myocyte's capacity to oxidize fatty acids, increases reactive oxygen species production, and represses oxidative metabolism genes, such as UCP3 and PGC‐1α, leading to a higher susceptibility to lipotoxicity. Muscle‐specific UCP3 transgenic mice have improved glucose homeostasis under chow and high‐fat diet (HFD) conditions, as well as resistance to obesity‐induced diabetes.^S36–S38^ Moreover, increased levels of circulating lipids increase UCP3 expression.^S39, S40^ We demonstrate that Nr2f6 is downregulated when C2C12 cells are exposed to fatty acids and when there is an increase in β‐oxidation in mice. This reduction may lift UCP3 transrepression, supporting the hypothesis that the downregulation of Nr2f6 is part of an adaptative response to lipid exposure.

Sarcopenia is the age‐related loss of muscle mass and function, sustained by reduction of the number and area of myofibres^S41^ and by mitochondrial dysfunction.^S42^ Contrasting with most NRs, Nr2f6 overexpression in muscle induces a sarcopenia‐like phenotype with muscle atrophy, hypoplasia, inflammation, reduced strength, and altered energy metabolism. Conversely, Nr2f6 knockdown in myotubes improves mitochondrial function and increases MHC expression, indicating that controlling Nr2f6 levels could be effective in treating sarcopenia and other myopathies, which is in line with recent bioinformatic analysis indicating Nr2f6 as a putative regulator of muscle energetic balance and development.^S43^ Nr2f6 agonists have been proposed for colitis treatment^S44^; however, in light of the considerable muscle waste in Nr2f6 gain‐of‐function muscles reported here, the use of such agonists for patients suffering from myopathies and cachexia should be further evaluated. Although the atrophic phenotype described here could be partially underlaid by the action of Nr2f6 in immune cells, the antagonistic transcriptional and functional changes induced by Nr2f6 overexpression *in vivo* and its knockdown *in vitro* in various experimental models strongly indicate a direct action of Nr2f6 in the myofibres as the major driver of the functional changes. A conceivable mechanistic model of Nr2f6‐induced force production impairment (Figure S4D), entails the downregulation of contraction‐related pathways, including muscle structure, calcium cycling, and action potential transmission. The ryanodine receptor 1 (*RYR1*) is a major component of the calcium release complex, which mediates calcium efflux from the sarcoplasmic reticulum into the cytosol and *in vivo* knockdown and mutations of *RYR1* lead to severe myopathies.^S45^ Other putative targets including myosin light chain kinase 4 (*MYLK4*) and myomesin 1 (*MYOM1*) are downstream of the androgen receptor (AR) and mediate its effects on force production.^S46^ The spermine oxidase gene (*SMOX*), another important target of the AR in muscle^S47, S48^ is downregulated by Nr2f6 overexpression and upregulated by Nr2f6 silencing. Therefore, possible functional associations between Nr2f6 and AR warrant further studies.

Nr2f6 can activate transcription by tethering to *circRHOT1*,^S49^ *DDA1*,^S50^ and *CD36* ^9^ promoters. Conversely, it represses the expression of numerous other genes such as *IL17*, *IL21*, renin, and oxytocin.^10^ ^,S22,S51,S52^ Our transcriptomics experiments display an equilibrated number of up and downregulated genes, suggesting that Nr2f6 is a dual‐function transcription factor. The case of CD36 illustrates this context‐dependent regulation as it is activated by Nr2f6 in the liver^9^ but repressed in skeletal muscle. This suggests that Nr2f6 activity could be modulated by post‐translational modifications or interaction partners, including miRNAs^S53^ and proteins, such as RAR related orphan receptor γ (RORγ), an Nr2f6 interactor that regulates *CD36* transcription in muscle^S54^ and liver.^S55^

In ovarian cancer, Nr2f6 promotes cell proliferation by tethering the histone acetylase P300 to the Notch3 promoter.^25^ We found that Nr2f6 induces myoblast proliferation, increases the expression of proliferation markers *in vivo*, and key regulators of the cell cycle, such as Cdk1 and its activator cyclin B1 (Ccnb1), are placed among high‐confidence Nr2f6 targets. Ccnb1 ectopic expression increases cell proliferation rates and it is upregulated in several types of cancer.^S56,S57,S58^ The simultaneous effect of Nr2f6 modulation on cell cycle and differentiation markers might also be sustained indirectly by RB1, which is responsible for cell cycle arrest through the inhibition of the E2F family of transcription factors. In a feedback loop, E2F transcription factors antagonize MyoD1, which induces RB1 expression, thereby linking the processes of proliferation and differentiation.^S59,S60^ While Nr2f6 increases the expression of myogenin in the *tibialis anterior* muscle, myosin heavy chains and other indicators of terminally differentiated myofibres are sharply reduced, sustaining the possibility that Nr2f6 overexpressing myoblasts proliferate but fail to assemble into robust myofibrils due to a dysregulated modulation of the MRFs during myogenesis. Nr2f6 ChIP‐seq peaks are associated with important transcriptional regulators of myogenesis, including GATA5, which regulates crucial genes for heart function.^S61,S62^ Also, ELK1 and FOS, which are downstream targets of ERK1/2, are necessary for proper muscle differentiation^S63–S65^ and regeneration,^S66,S67^ respectively. Interestingly, affinity capture‐MS experiments identified FOS as an Nr2f6 interactor.^S68^ Using different approaches, we demonstrate that Nr2f6 is part of a cell cycle regulatory network and partners with other transcription factors to modulate muscle cell differentiation and proliferation.

The evidence gathered here points to a global regulation of pivotal aspects of skeletal muscle biology by Nr2f6, through a concerted control of multiple gene networks. Nr2f6 modulation alone can determine myoblast proliferation rates, consolidating its role as a general regulator of cell cycle progression in different lineages, and, in the skeletal muscle, acting as a fulcrum between myogenesis and cell division. Conversely, our findings amount to the hypothesis that the metabolic outcomes of Nr2f6 modulation are tissue specific. Our discoveries establish Nr2f6 as a novel regulator of muscle contraction and metabolism and allude to a potential therapeutic strategy for muscle wasting and metabolic diseases.

## Conflict of interest

The authors declare that they have no conflict of interest.

## Supporting information


**Figure S1.** Nr2f6 regulates myogenesis and binds to the promoters of genes involved in metabolism in different cell types. (A) Validation of Nr2f6 knockdown in siScr and siNr2f6 transfected C2C12 myotubes by RT‐qPCR (leftmost) and western blot (rightmost). (B) Volcano plot of Nr2f6 knockdown C2C12 myocytes. Genes upregulated in red and downregulated in blue (FDR < 0.05) (*N* = 4–5). (C) Correlation of differentially expressed genes in the transcriptome of siNr2f6 myocytes and public C2C12 differentiation microarray (GSE4694). (D) Manually selected insulin signalling pathway schematic displaying differentially expressed genes after Nr2f6 knockdown and other components of the pathway. Metabolites are depicted in yellow borders and unchanged genes are in orange borders. Fold‐change and FDR level of depicted genes in the RNA‐seq are shown in the table.
**Figure S2.** Nr2f6 depletion enhances metabolism in skeletal muscle. (A, B) Oxygen consumption assay in C2C12 myocytes transfected with siScr (control) and siNr2f6. On the right, are the calculated metabolic parameters. (C) Oligomycin‐induced extracellular acidification rate during a high‐glucose oxygen consumption assay (*N* = 4). (D) Lactate measurement in cell culture media of C2C12 myocytes transfected with control siScr and siNr2f6 (*N* = 3). (E) ATP content in siScr and siNr2f6 transfected myocytes (*N* = 3). (F) Cell death as measured by propidium iodide in control (shGFP) and shNr2f6 myocytes following treatment with 500 μM palmitate for 20 hours (N = 3). (G, H) Body weight and glucose tolerance test of mice undergoing 16 weeks of a high‐fat diet. (I) Relative Nr2f6 mRNA expression in the gastrocnemius of mice fed with a control chow diet or high‐fat diet (HFD) for 16 weeks (*N* = 7). (J) Gene ontology analysis of genes with Nr2f6 binding sites within ±3 kbp of the transcription start site in both K562 and HepG2 ChIP‐seq datasets from the ENCODE project. (K) Enrichment of KEGG pathways terms of the upregulated (left) and downregulated (right) kinases in the siNr2f6 transcriptome. Data displayed as mean ±SD. Boxplot with whiskers spanning minimum to maximal and box edges 25th–75th percentile, line at the median, and + at the mean. * Indicates *p* < 0.05 using unpaired two‐tailed Student's t‐test.
**Figure S3.** Nr2f6 regulates UCP3 and PGC‐1α expression. (A) Gene expression was measured by RT‐qPCR in primary mouse skeletal muscle cells transfected with siScr (control) and siNr2f6 (*N* = 3). (B) Luciferase reporter assay of the responsive elements of the Oestrogen Related Receptor (ERRE) and PPAR (PPRE) in MEF cells transfected with siScr or siNr2f6 (*N* = 5). (C, D) Gene expression (N = 3) and representative western blot for validation of Nr2f6‐myc stable myotubes. Boxplot with whiskers spanning minimum to maximal and box edges 25th–75th percentile, the line at the median and + at the mean. * Indicates *p* < 0.05 using unpaired two‐tailed Student's t‐test. (E) Mouse UCP3 genomic locus retrieved from UCSC Genome Browser with the Nr2f6 response element highlighted. Top tracks: ChIP‐seq of Myogenin and MyoD at 24 h and 60 h of differentiation. Middle track: DNAse hypersensitivity assay, with open sensitive regions in grey. Bottom tracks: histone marks ChIP‐seq. (F) Mouse PGC‐1α genomic locus with the Nr2f6 response element highlighted in yellow and the region cloned in the reporter plasmid in grey.
**Figure S4.** Nr2f6 overexpression impairs muscle function. (A) Heat‐map of top 30 most modulated genes in tibialis anterior muscle electroporated with control empty vector (EV) or an Nr2f6‐myc‐coding plasmid (*N* = 4). (B) Atrogenes regulated by Nr2f6 overexpression in the tibialis anterior muscle. * Denotes significant modulation (FDR < 0.05, fold‐change > 2) in the microarray. (C) Time to fatigue in ex vivo contraction was set as the necessary time to reach 50% of the maximal force with constant stimulation (*N* = 6). (D) Nr2f6 overexpression reduces the expression of several genes of the contractile apparatus, myofiber calcium handling, and action potential transduction. Genes with Nr2f6 binding motif at the promoter are underscored. Differentially expressed genes following Nr2f6 overexpression in mouse TA were selected according to ontology terms related to muscle contraction and function. The arrows indicate the up‐ or downregulation. Sodium Voltage‐Gated Channel Alpha Subunit 4 (Scn4a), Potassium Inwardly Rectifying Channel Subfamily J Member 2 (Kcnj2), Solute Carrier Family 8 Member A3 (Slc8a3), Muscle Associated Receptor Tyrosine Kinase (Musk), Ryanodine Receptor 1 (Ryr1), Calsequestrin 1 (Casq1), ATPase Sarcoplasmic/Endoplasmic Reticulum Ca2 + Transporting 2 (SERCA2, Atp2a2), Cholinergic Receptor Nicotinic Alpha 1/delta/gamma subunit Chrna1/d/g), Troponin T1/I1/I2/C1 (Tnnt1/Tnni1/Tnni2/c1), Myom1/2 (Myomesin1/2), Myozenin1/3 (Myoz1/3), Myosin light chain kinase 2/4 (Mylk2/4), Myosin heavy chain 3 (Myh3), Myosin binding protein C1/2 (Mybpc1/2).
**Figure S5.** Increased Nr2f6 expression in aged muscle. (A) Nr2f6 expression in muscles of healthy donors and bearers of hereditary spastic paraplegia (HSP) and juvenile dermatomyositis (JDM) from public datasets, GSE3307 and GSE11971, respectively. (B) Gastrocnemius muscles of 3‐month‐old (Young) and 18‐month‐old (Old) mice were collected and processed for RT‐qPCR as described elsewhere (*N* = 7–9). Boxplot with whiskers spanning minimum to maximal and box edges 25th–75th percentile, the line at the median and + symbol at the mean. p‐values < 0.05 depicted as *.
**Figure S6.** (A) Scatter plot of differentially expressed genes in Nr2f6 knockdown in C2C12 myocytes RNA‐seq (FDR < 0.05) and Nr2f6 overexpression in mice TA microarray (FDR < 0.05, fold‐change >2). In red: genes upregulated by Nr2f6; in blue: genes downregulated by Nr2f6; in grey: genes with the same direction of modulation by Nr2f6 overexpression and knockdown. (B) Quantitation of the indicated proteins by western blot in *tibialis anterior* muscles electroporated with empty vector (EV) in the lateral leg or Nr2f6‐myc plasmid in the contralateral leg (*N* = 4). Individual samples are depicted as circles and * indicates *p* < 0.05 using ratio paired two‐tailed Student's t‐tests, when larger than 0.05, p‐values are indicated above comparisons.
**Figure S7.** Bioinformatic analyses of Nr2f6 ChIP‐seq Nr2f6 peak regions from HepG2 and K562 cells were retrieved from the UCSC genome browser, and the overlapping sequences were extracted. *De novo* motif enrichment was performed in the resulting sequences and the corresponding most similar transcription factor motif was attributed. (A) Workflow of bioinformatic analysis of Nr2f6 ChIP‐seq data. (B) Top 10 largest fold‐change values in siNr2f6 RNA‐seq that contain an Nr2f6 binding peak in both ChIP‐seq experiments. (C) Gene ontology of the unique genes regulated by Nr2f6 knockdown. (D) De novo motif analysis of Nr2f6 binding regions conserved in both ChIP‐seq data. (E) Transcription factors are most likely represented by the lowest E‐value matrix.

**Table S1.** List of primers and TaqMan probes used for gene expression.

**Table S2.** Key resource table.
