# Supplementary material for: Concerted regulation of skeletal muscle metabolism and contractile properties by the orphan nuclear receptor Nr2f6
Source: J Cachexia Sarcopenia Muscle. 2024 Apr 29;15(4):1335–47. doi: 10.1002/jcsm.13480 (PMC11294040; doi:10.1002/jcsm.13480)
Supplement: Supplementary file 1 — Figure S1. Nr2f6 regulates myogenesis and binds to the promoters of genes involved in metabolism in different cell types. (A) Validation of Nr2f6 knockdown in siScr and siNr2f6 transfected C2C12 myotubes by RT‐qPCR (leftmost) and western blot (rightmost). (B) Volcano plot of Nr2f6 knockdown C2C12 myocytes. Genes upregulated in red and downregulated in blue (FDR < 0.05) (N = 4–5). (C) Correlation of differentially expressed genes in the transcriptome of siNr2f6 myocytes and public C2C12 differentiation microarray (GSE4694). (D) Manually selected insulin signalling pathway schematic displaying differentially expressed genes after Nr2f6 knockdown and other components of the pathway. Metabolites are depicted in yellow borders and unchanged genes are in orange borders. Fold‐change and FDR level of depicted genes in the RNA‐seq are shown in the table. Figure S2. Nr2f6 depletion enhances metabolism in skeletal muscle. (A, B) Oxygen consumption assay in C2C12 myocytes transfected with siScr (control) and siNr2f6. On the right, are the calculated metabolic parameters. (C) Oligomycin‐induced extracellular acidification rate during a high‐glucose oxygen consumption assay (N = 4). (D) Lactate measurement in cell culture media of C2C12 myocytes transfected with control siScr and siNr2f6 (N = 3). (E) ATP content in siScr and siNr2f6 transfected myocytes (N = 3). (F) Cell death as measured by propidium iodide in control (shGFP) and shNr2f6 myocytes following treatment with 500 μM palmitate for 20 hours (N = 3). (G, H) Body weight and glucose tolerance test of mice undergoing 16 weeks of a high‐fat diet. (I) Relative Nr2f6 mRNA expression in the gastrocnemius of mice fed with a control chow diet or high‐fat diet (HFD) for 16 weeks (N = 7). (J) Gene ontology analysis of genes with Nr2f6 binding sites within ±3 kbp of the transcription start site in both K562 and HepG2 ChIP‐seq datasets from the ENCODE project. (K) Enrichment of KEGG pathways terms of the upregulated (left) and downr [file JCSM-15-1335-s003.docx]

**Supplementary Information for**

Concerted regulation of skeletal muscle metabolism and contractile properties by the orphan nuclear receptor Nr2f6

Dimitrius Santiago P.S.F Guimarães^#^, Ninon M.F. Barrios, André Gustavo de Oliveira, David Rizo-Roca, Maxence Jollet, Jonathon A.B. Smith, Thiago Reis Araújo, Marcos Vinicius da Cruz, Emilio Marconato Junior, Sandro M. Hirabara, André S. Vieira, Anna Krook, Juleen R.^,^ Zierath, Leonardo R. Silveira

^#^Corresponding author

**Email:**  dimitrius.guimaraes@ki.se; d211529@dac.unicamp.br

Contents:

Supplementary Methods

Supplementary Figures 1 – 7

References (continuation)

**Supplementary Methods**

**2.1. Cell culture** Human primary skeletal muscle cells were isolated from healthy female and male donors^13^, 55 ±5 years old, BMI 25.6 ±1.5 kg.m^-2^. Myoblasts were maintained in Growth Media (Dulbecco's Modified Eagles Media (DMEM)/F12 High Glucose (Gibco, #31331093) supplemented with 10 mM HEPES (Gibco #15630-056), 16% Fetal calf serum (Sigma, #F7524), and antibiotics (Gibco #15240-062) and differentiated at the confluence with fusion media (74% DMEM High Glucose (Gibco, 31966-021), 20% 199 Medium (Gibco #31150-022), 20 mM HEPES, antibiotics, 0.03 µg/mL Zinc Sulfate (Sigma #Z4750), 1.4 mg/mL Vitamin B12 (Sigma #V6629), and 2% Fetal Calf Serum) supplemented with 100ug/mL Apotransferrin (Biotechne #3188-AT-001G) and 1.7 mM Insulin (Actrapid Penfill, Novo Nordisk #13509) before use. After 5 days of fusion, apotransferrin and insulin were removed, and cells were incubated for 4 more days. Cells were cultivated in a humidified atmosphere containing 7.5% CO2 and regularly tested for mycoplasma. C2C12s, MEFs, and HEK cells were maintained in DMEM High Glucose (Gibco, 31966-021) supplemented with 4 mM L-glutamine, 10% fetal bovine serum, 1 mM sodium pyruvate, and antibiotics. Fetal bovine serum was substituted by 2% horse serum to induce myogenesis in C2C12 cells when 90-100% confluence was reached, and experiments were performed 5 days later in fully differentiated myotubes.

**2.2. Primary mouse skeletal muscle cells** **Mouse** primary skeletal muscle cells were isolated from wild-type C57Bl6/JUnib as described^14^. After euthanasia, hindlimb muscles were dissected and digested with collagenase II, trypsin, and DNAse I. Cells were sifted through a 70 µm cell strainer and plated in 0.1% Matrigel-coated plates. Myoblasts were maintained for 2 days in DMEM High Glucose supplemented with 2 mM L-glutamine, 10% fetal bovine serum, 10% horse serum, 1 mM sodium pyruvate, and antibiotics. Myogenesis was induced by removing fetal bovine serum from the media when total confluence was reached, and cells were cultivated for 5 more days to form fully differentiated myotubes. The experiments were approved by the Ethics Committee on Animal Use (CEUA/Unicamp #5626-1/2020).

**2.3. Animals** All electroporation experiments were conducted following the guidelines of animal welfare and were approved by the Stockholm North Animal Ethical Committee (Stockholm, Sweden). Male C57Bl6/J mice were acquired from Jackson Labs and maintained at 12/12h light/dark cycle under controlled temperature and humidity, and *ad libitum* access to food (Specialized Research Diets, # 801722) and water. The use of animals for high-fat diet experiments was approved by the Ethics Committee on Animal Use (CEUA/Unicamp #5626-1/2020) and all the welfare guidelines of the National Council of Control of Animal Experimentation (CONCEA) were followed. Male C57Bl6/JUnib mice were kept under the same conditions described above. Mice were provided a high-fat diet (PragSolucoes #0015, 60% kcal from lipids) at 4 weeks of age for 16 weeks; littermates were fed a standard chow diet as a control.

**2.4. Reactive oxygen species measurement** Cells were incubated with 5 nM MitoSOX (Invitrogen, #M36008) or 5 µM Dihydroethidium (DHE, Invitrogen, #D11347) for 30 min in DMEM without phenol red supplemented with 1 mM Sodium Pyruvate, 4 mM L-glutamine, and 25 mM Glucose and washed three times before reading in a plate reader 510/580 nm (ex./em.) or 520/610 nm (ex./em.) for MitoSOX and DHE, respectively. For normalization, samples were immediately fixed with 5% formaldehyde, stained with 0.05% Crystal Violet solution for 15 minutes, and thoroughly washed with water. The dye was resuspended in 10% acetic acid and absorbance read at 590 nm in a plate reader.

**2.5. RT-qPCR** Total RNA was extracted from cells with TRIzol (Invitrogen #15596-018) following the manufacturer’s instruction and cDNA was synthesized with a High-Capacity Reverse Transcription kit (Applied Biosystems #4368814). cDNA was diluted to 10 ng/µL and 20 ng was used for qPCR reactions. NormFinder^15^ was used to decide the best combination of internal controls among RPL39, PPIA, HPRT, 18S, ACTB, and GAPDH. In the *in vivo* electroporation experiments, gene expression was normalized using HPRT-PPIA geomean with TaqMan probes or HPRT-RPL39 geomean when SYBER green was used. For other experiments, gene expression was normalized with multiplexed HPRT when TaqMan probes were used or with RPL39 when SYBER was used. Relative gene expression was calculated by the ∆∆C_T_ method^16^ and is expressed as fold change over the indicated control. The primers and probes used are listed in Supplementary Table 2.

**2.6. RNA-seq** Total RNA was extracted with TRIzol and the upper phase containing RNA was loaded into RNeasy columns (Qiagen, #74004) after the addition of isopropanol, following the manufacturer’s instructions. cDNA libraries were prepared with TruSeq Illumina Total RNA Stranded (Illumina) with Ribo-zero rRNA depletion (Illumina). Sequencing was outsourced to Macrogen Inc. (Seoul, South Korea) and performed in a HiSeq X (Illumina), producing an average of 50.8 Mreads, 95% above Q30. Sequence trimming and adapter removal were done with Trimmomatic^17^ with the following modifications: HEADCROP = 10, MINLEN = 20, AVGQUAL = 20. Reminiscent reads were aligned to the mouse genome (Ensembl GrCm38) with RNA Star 2.7.2b and gene-level counts were calculated with featureCounts v1.6.4. Differential expression was performed with EdgeR with TMM normalization and p-value adjustment for multiple comparisons using Benjamini and Hochberg normalization with a 0.05 false discovery rate (FDR) cut-off. The Galaxy platform was used to process all data. Pathway enrichment analysis was done in g:Profiler with a 0.01 FDR cutoff. Interaction networks were generated by String.db and analyzed with CytoScape v3.8 using the EnrichmentMap plugin.

**2.7. Fatty-acid treatment** Palmitate (Sigma, #P5585) in absolute ethanol

and oleate (Sigma, #O7051) in water was conjugated with 1% fatty-acid-free bovine serum albumin (Sigma, #A7030) in cell media for 15 min at 55 ^o^C to a 500 µM final concentration. Cells were treated with fresh solutions of palmitate or vehicle (1%BSA, 1% ethanol) for 20 hours.

**2.8. Promoter transactivation assays** Luciferase reporter assays were performed in MEF cells transfected with a UCP3 reporter plasmid^18^ (UCP3 EP1, Addgene #71743) or PGC-1α 2kb promoter (Handschin et al. 2003) (Addgene #8887), normalization plasmid coding for Renilla luciferase (pRL-SV40) and either control empty vector or Nr2f6 coding plasmid (Gift from Dr. Gottfried Baier, Medical University of Innsbruck, Austria) using Lipofectamine 3000. Luciferase activity was measured with DualGlo Luciferase Reporter Assay (Promega, #E2920). For knockdown assays, cells were transfected with siRNAs one day before the transfection of the reporter plasmids.

**2.9. siRNA knockdown** C2C12 cells were transfected with 200 nM non-target siRNA (siScr, Qiagen) or siNr2f6 (Sigma) using Lipofectamine RNAiMax (Invitrogen) following manufacturer’s instructions concomitantly with the differentiation media switch. Experiments were performed on the third day of differentiation. Primary human skeletal muscle cells were double transfected, first concomitantly with the fusion media switch and later on the third day of differentiation with 5 nM siScr (Ambion) or siNr2f6 (Ambion). The experiments were performed on the seventh day of differentiation.

**2.10. Stable cell lines** The Nr2f6-myc insert was subcloned from the Nr2f6-myc-flag plasmid into the pBABE-Puro vector using standard PCR with primers spanning the transcription start site and the myc tag. The viral particles for generating the overexpression HEK293T cells were transfected with pCMV-VSVG, pCL-Eco, and the pBABE-Nr2f6-myc or empty vector. For producing viral particles with the knockdown plasmid, HEK293T cells were transfected with packaging vectors pCMV-dR8.2 dvpr, pCMV-VSVG, and pLKO.1-shGFP or shNr2f6 (TRCN0000026147). Cell medium containing virions was collected, filtered at 0.45 µm, and stored at -80 oC until further use. Virus concentrations were titrated by the minimal dilution method, C2C12 cells were transduced with 1 MOI, and cells were selected with 2 µg/mL puromycin for 4 days. The clonal selection was performed in the knockdown cells and the clones were validated as indicated. The modified cells and their respective controls were cultivated synchronously under the same conditions.

**2.11. Electroporation** Mice were kept under 2% isoflurane-induced anesthesia and the tibialis anterior muscles were injected with 30 µL of 1 mg/mL hyaluronidase (Sigma, #H3506). After 2 hours, the lateral and contralateral tibialis anterior were injected with 30 µg of either control empty vector pCMV6 or Nr2f6-myc-flag overexpression plasmid (Origene, #MR206083) and 220V/cm were applied in 8 pulses of 20/200 ms on/off (ECM 830 Electroporator, BTX). Terminal experiments were performed 9 days after electroporation with 13-week-old mice. For electroporation of FDB muscles, after anesthesia 10 µL of 1 mg/mL hyaluronidase was injected into the footpads and after 1 hour, 20 µg of the control or Nr2f6 coding plasmids. Mice rested for 15 minutes and then 75V/cm was applied in 20 pulses of 20/99 ms on/off with the aid of sterile gold acupuncture needles.

**2.12. *Ex vivo* contraction** Mouse *flexor digitorum brevis* (FDB) muscles were electroporated as described and dissected 8 days later. To reduce variability dissections were performed by a single trained technician. With the muscles still attached to the tendons, contraction threads were tied at the most distal and proximal tendons, and the muscles were transferred to contraction chambers (Myograph system, DMT A/S) containing prewarmed and continuously oxygenated KHB buffer at 30 ^o^C. The optimal muscle length was determined, and all subsequent measurements were performed at this length. For maximal force production mice FDBs were stimulated at 10, 30, 50, 80, 100, and 120 Hz for 1 second and with 0.1 ms pulses. Muscles were left to rest for 5 minutes before starting the fatigue protocol as follows: 0.1 s train duration, 0.3 s train delay, and pulses of 0.1 ms at 50 Hz. The maximal force was evaluated again 5 min after the end of the fatigue protocol to check muscle integrity. Muscles were weighed and protein extraction was performed to normalize. The maximal force was calculated with the difference of the peak force at 120 Hz and the baseline and time to fatigue taken as the time necessary to reach 50% intensity of the first peak.

**2.13. MHC Staining** Electroporated tibialis anterior muscles were embedded in O.C.T, immediately frozen in nitrogen-cooled isopentane, and stored at -80 ^o^C until cryosectioning. Muscle slices were blocked (5% Goat serum, 2% BSA, 0,1% sodium azide in PBS) for 3 hours at room temperature and probed with primary antibodies overnight at 4 ^o^C in a humidified chamber. The slides were washed 3 times with PBS and incubated with Alexa Fluor conjugated secondary antibodies (Invitrogen) for 2 hours at room temperature. Coverslips were mounted with ProLong antifade Diamond (Invitrogen) and whole sections were imaged with a fluorescent scanning microscope (AxioScan.Z1 Slide Scanner, Zeiss) at 20x magnification. Controls without primary antibodies were used to calibrate acquisition parameters and for image analysis. For fiber type quantitation, the images were randomized, and the analysis was performed blinded to the treatments, at least 2 consecutive cuts were quantified and averaged for each sample group in a given mice.

**2.14. Oxygen consumption assays** Oxygen consumption rates (OCR) were measured in a Seahorse XF24 extracellular flux analyzer according to the manufacturer’s instructions. The following drugs were used in the assay: 1 μM oligomycin (Oligo), 2 μM carbonyl cyanate m-chlorophenyl hydrazone (CCCP), and 1 μM rotenone/antimycin (Rot./Ant). ATP-linked OCR was calculated by subtracting OCR post oligomycin addition from the OCR measured before. Reserve capacity was determined by subtracting basal from maximal OCR. Non-mitochondrial values were subtracted before all calculations. For fatty-acid oxidation assays, cell media was switched to low glucose 12 hours before the measurements, and cells were equilibrated in KHB supplemented with 1g/L glucose, 4 mM L-glutamine, and 1 mM sodium pyruvate for 1 hour. Immediately before the assay, BSA-conjugated palmitate was added to a final concentration of 200 µM, and the drugs were added in the same manner. During routine oxygen consumption assays, cells were maintained in phenol red-free DMEM, supplemented with 4.5g/L glucose, 4 mM L-glutamine, and 1 mM sodium pyruvate, without sodium bicarbonate.

**2.15. Lactate measurement** Cells were grown in 96well plates and then incubated for 3 h with 50 µL Krebs-Henseleit Buffer (KHB) (1.2 mM Na2HPO4, 2 mM MgSO4, 4.7 mM KCl, 111 mM NaCl, pH 7.3) supplemented with 25 mM glucose, 1 mM pyruvate, and 4 mM Glutamine. Lactate production was enzymatically quantified as NADH fluorescence (360 nm/460 nm) by the reverse reaction of L-lactate dehydrogenase (Rabbit muscle, L25005KU, Sigma) in a reaction containing 20 µL cell media, 2 µg enzyme, 50 mM Tris, and 625 mM Hydrazine in PBS. Following the assay, the cells were fixed and stained with crystal violet for cell number normalization.

**2.16. Western blot** Protein extracts from cells and tissues were obtained with RIPA Buffer (Thermo Scientific, # 89900). Briefly, cells were collected and homogenized in RIPA buffer with the aid of a sonicator, incubated on ice, and centrifuged at 16000 xg for 20 minutes to remove insoluble materials. Protein in the supernatant was determined using Bradford assay and 30 µg loaded into 4-10% or 4-12% gradient SDS-PAGE gels (Mini-PROTEAN TGX Precast Gels, **Bio-Rad**). Proteins were then transferred to 0.45 µm PVDF membranes (Immobilon-P, Millipore) in a wet tank apparatus, probed with the indicated primary antibodies, and detected with ECL (ECL Select, Cytiva #RPN2235) in a ChemiDoc XR (**Bio-Rad**). Images were analyzed with ImageLab software (**Bio-Rad**) and protein band intensities were normalized by the Ponceau S intensity of the respective gel lane. Data is shown as fold change over control.

**2.17. Microarray** RNA was extracted with TRIzol and subsequently column-purified using RNeasy Mini Kit (Qiagen). Sample integrity was assessed, and the library was prepared using an Affymetrix Whole Transcript (WT) Assay kit probed in a CGAS cartridge for Clariom S (mouse) following the manufacturer’s instructions. Total RNA quality was assessed by Agilent Technologies 2200 Tapestation and concentrations were measured by NanoDrop ND-1000 Spectrophotometer. Total RNA (150 ng) was used to generate amplified sense strand cDNA targets using GeneChip WT Plus Reagent Kit (ThermoFisher Scientific) followed by fragmentation and labeling. 2.3 µg of ss cDNA target was hybridized to Clariom S Mouse Arrays for 16 hours at 45 °C under rotation in Affymetrix Gene Chip Hybridization Oven 645 (ThermoFisher Scientific). Washing and staining were carried out on Affymetrix GeneChip Fluidics Station 450 (ThermoFisher Scientific), according to the manufacturer’s protocol. The fluorescent intensities were determined with Affymetrix GeneChip Scanner 3000 7G (ThermoFisher Scientific). Transcriptome Analysis Console (TAC) software (v4.0.3, ThermoFisher Scientific) was used for the analysis of microarray data. Signal values were log2-transformed, and quantile normalized using the Signal Space Transformation (SST-RMA) method. Since control and treated samples were obtained from the same animal, paired comparisons of gene expression levels between sample groups were performed using a moderated t-test as implemented in BioConductor package limma. Genes with FDR < 0.05 and fold change ≥ 2 were considered differentially expressed Gene ontology enrichment tests were performed with g:profiler excluding electronic annotations and using a significance threshold of 0.01.

**2.18. Cell-death assays** Cell-death assays were performed as described ^19^ with slight modifications. Propidium iodide (Invitrogen #P3566) was added to a concentration of 5 µg/mL in cell culture media and incubated for 20 minutes. Hoechst 33342 (Invitrogen #H3570) was then added to a final concentration of 1 µg/mL and samples were incubated for another 10 minutes. Fluorescence was measured at 530/620 nm (ex./em.) and 350/460 (ex./em.) nm in a plate reader.

**2.19. Cell doubling time** Cells (10^4^) were plated in four replicates in 12-well plates. Thereafter, cells were collected every 24 hours using trypsin and counted in a Neubauer chamber. The normalized data of three independent experiments were used to obtain the doubling-time regression curve with the initial number constraint.

**2.20. ATP measurement** Cells were grown in opaque 96-well white plates and then processed according to the manufacturer’s instructions of the CellTiter-Glo Luminescent Cell Viability Assay kit (Promega # G9241). The standard curve of ATP was determined in parallel for absolute quantitation.

**2.21. Bioinformatic analysis of public datasets** **Nr2f6 ChIP-seq in HepG2 and K562 cells from the ENCODE project available on GEO (Gene Expression Omnibus) accession codes GSM2797593 and GSM2534343^20,21^ were used. Peak coordinates and sequences were retrieved from the UCSC table browser and filtered with a 0.02 log_10_Qvalues cutoff. Using bedtools in the Galaxy platform, the coordinates of overlapping sequences were retrieved and annotated with ChIPseeker considering a promoter region of ±3 kbp from the transcription start site.** Pathway enrichment was analyzed using the g.profiler program (https://biit.cs.ut.ee/.profiler/gost), with a significance threshold of 0.01 using the g:SCS parameter, without considering electronic term annotations. The correlation in Supplementary Figure 1C was produced with fold changes of differentially expressed genes from RNA-seq (FDR <0.05) and fold change values (expression in myotube/expression in myoblast) from the C2C12 cell differentiation array (GSE4694) ^22^ considering a p-value cut-off of 0.01, according to GEO2R. UCP3 genome locus in Supplementary Figure 3E was extracted from the UCSC genome browser with the ChIP-seq tracks of Myogenin (wgEncodeEM002136, wgEncodeEM002132), MyoD (wgEncodeEM002127, wgEncodeEM002129), H3K4me (wgEncodeEM001450), H3K27Ac (wgEncodeEM001450) and DNA hypersensitivity track (wgEncodeEM003399) over NCBI37/mm9 mouse genome assembly. Nr2f6 response elements search (Supplementary Figure 3E and Figure 6A) was performed using JASPAR motifs MA0677.1, MA0728.1, and MA1539.1 in RSAT matrix scanning with background estimated from the input sequence and 10^-5^ p-value cutoff.

**2.22. Statistical analysis and quantification** GraphPad Prism v7.0 was used for plotting and statistical analysis. Cell culture experiments were performed independently several times with at least 3 technical replicates in each independent experiment. Given the cells of individual donors of human skeletal muscle cells are kept independently and each represents a single person, comparisons were made within the same donor using ratio paired Student’s t-test. The same principle was used for electroporation experiments, in which control and treated samples came from the same mice. Otherwise, unpaired comparisons were performed, and a 0.05 p-value significance cutoff was used. Before all comparisons, data normality was confirmed by the Shapiro-Wilk test. Details for microarray and RNA-seq statistics are described in their respective methods section and further statistical details are provided in figure labels.

**Supplementary Figures**
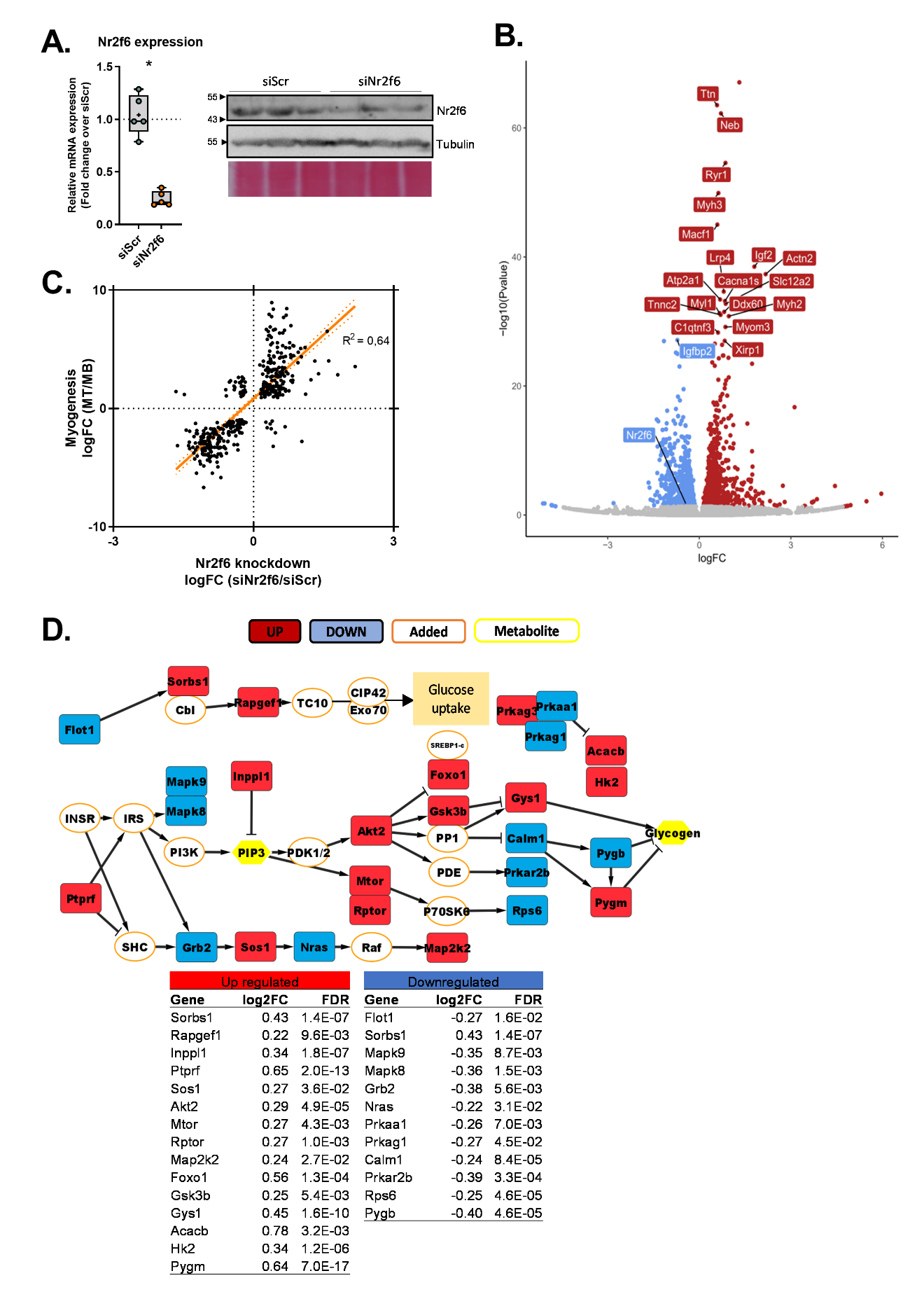


Fig. S1. Nr2f6 regulates myogenesis and binds to the promoters of genes involved in metabolism in different cell types. (A) Validation of Nr2f6 knockdown in siScr and siNr2f6 transfected C2C12 myotubes by RT-qPCR (leftmost) and western blot (rightmost). (B) Volcano plot of Nr2f6 knockdown C2C12 myocytes. Genes upregulated in red and downregulated in blue (FDR <0.05) (N = 4-5). (C) Correlation of differentially expressed genes in the transcriptome of siNr2f6 myocytes and public C2C12 differentiation microarray (GSE4694). (D) Manually selected insulin signaling pathway schematic displaying differentially expressed genes after Nr2f6 knockdown and other components of the pathway. Metabolites are depicted in yellow borders and unchanged genes are in orange borders. Fold-change and FDR level of depicted genes in the RNA-seq are shown in the table.


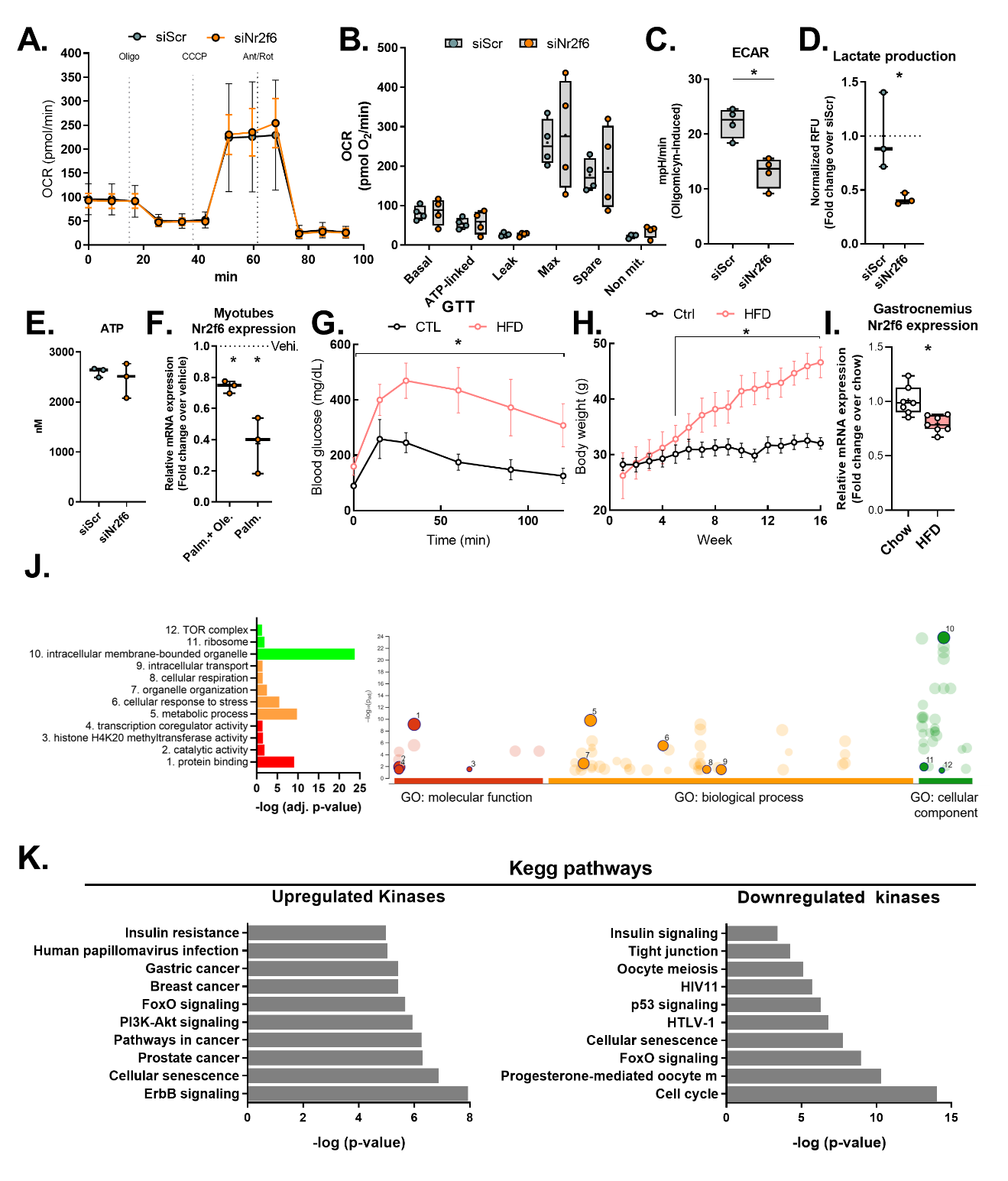


Fig. S2. Nr2f6 depletion enhances metabolism in skeletal muscle. (A, B) Oxygen consumption assay in C2C12 myocytes transfected with siScr (control) and siNr2f6. On the right, are the calculated metabolic parameters. (C) Oligomycin-induced extracellular acidification rate during a high-glucose oxygen consumption assay (N = 4). (D) Lactate measurement in cell culture media of C2C12 myocytes transfected with control siScr and siNr2f6 (N = 3). (E) ATP content in siScr and siNr2f6 transfected myocytes (N = 3). (F) Cell death as measured by propidium iodide in control (shGFP) and shNr2f6 myocytes following treatment with 500 µM palmitate for 20 hours (N = 3). (G, H) Body weight and glucose tolerance test of mice undergoing 16 weeks of a high-fat diet. (I) Relative Nr2f6 mRNA expression in the gastrocnemius of mice fed with a control chow diet or high-fat diet (HFD) for 16 weeks (N = 7). (J) Gene ontology analysis of genes with Nr2f6 binding sites within ±3 kbp of the transcription start site in both K562 and HepG2 ChIP-seq datasets from the ENCODE project. (K) Enrichment of KEGG pathways terms of the upregulated (left) and downregulated (right) kinases in the siNr2f6 transcriptome. Data displayed as mean ±SD. Boxplot with whiskers spanning minimum to maximal and box edges 25th-75th percentile, line at the median, and + at the mean. * Indicates p < 0.05 using unpaired two-tailed Student’s t-test.

**
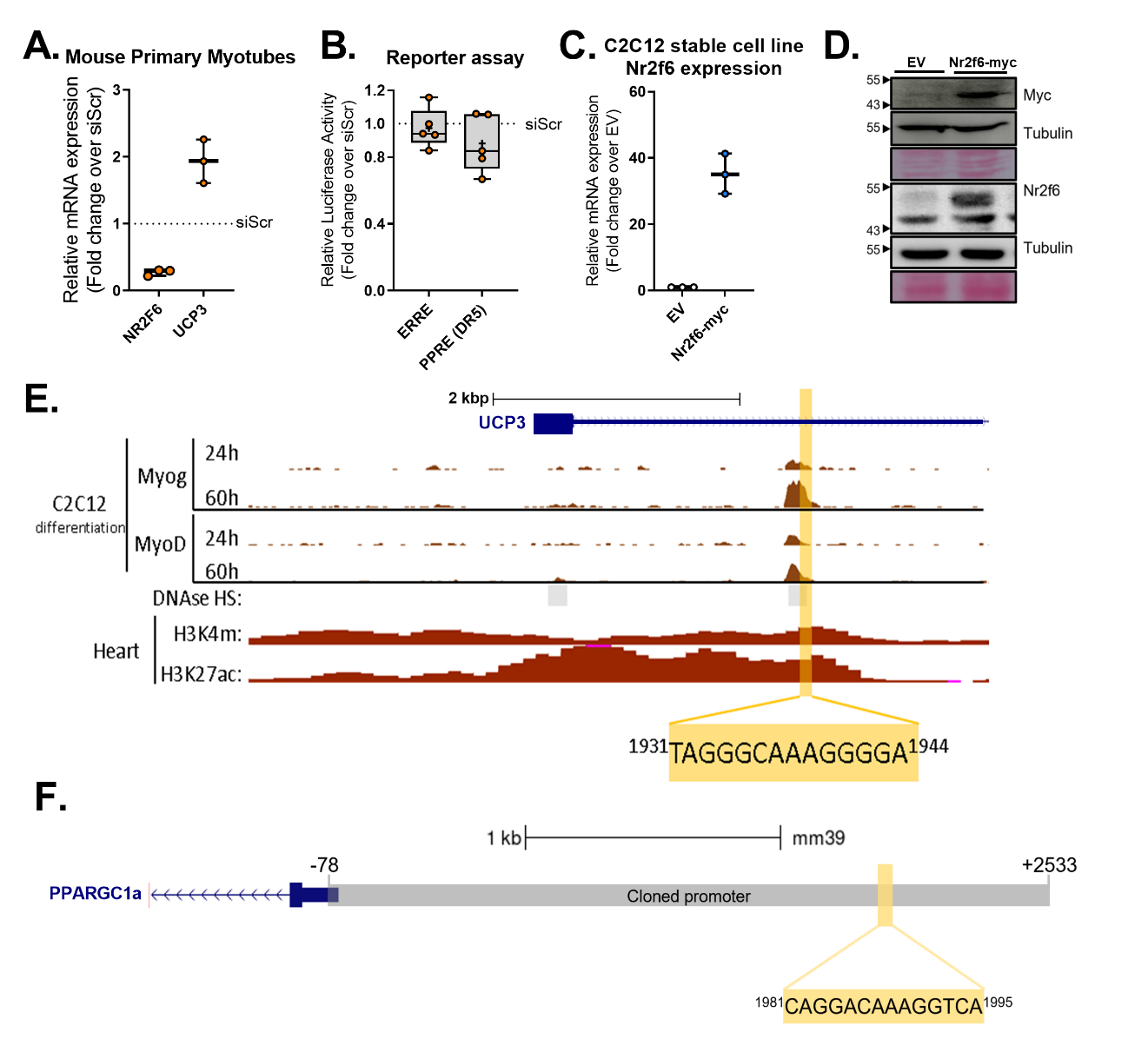
**

**Fig. S3. Nr2f6 regulates UCP3 and PGC-1α expression.** . (A) Gene expression was measured by RT-qPCR in primary mouse skeletal muscle cells transfected with siScr (control) and siNr2f6 (N = 3). (B) Luciferase reporter assay of the responsive elements of the Estrogen Related Receptor (ERRE) and PPAR (PPRE) in MEF cells transfected with siScr or siNr2f6 (N = 5). (C, D) Gene expression (N = 3) and representative western blot for validation of Nr2f6-myc stable myotubes. Boxplot with whiskers spanning minimum to maximal and box edges 25th-75th percentile, the line at the median and + at the mean. * Indicates p < 0.05 using unpaired two-tailed Student’s t-test. (E) Mouse UCP3 genomic locus retrieved from UCSC Genome Browser with the Nr2f6 response element highlighted. Top tracks: ChIP-seq of Myogenin and MyoD at 24h and 60h of differentiation. Middle track: DNAse hypersensitivity assay, with open sensitive regions in grey. Bottom tracks: histone marks ChIP-seq. **(F) Mouse PGC-1α genomic locus with the Nr2f6 response element highlighted in yellow and the region cloned in the reporter plasmid in grey.**


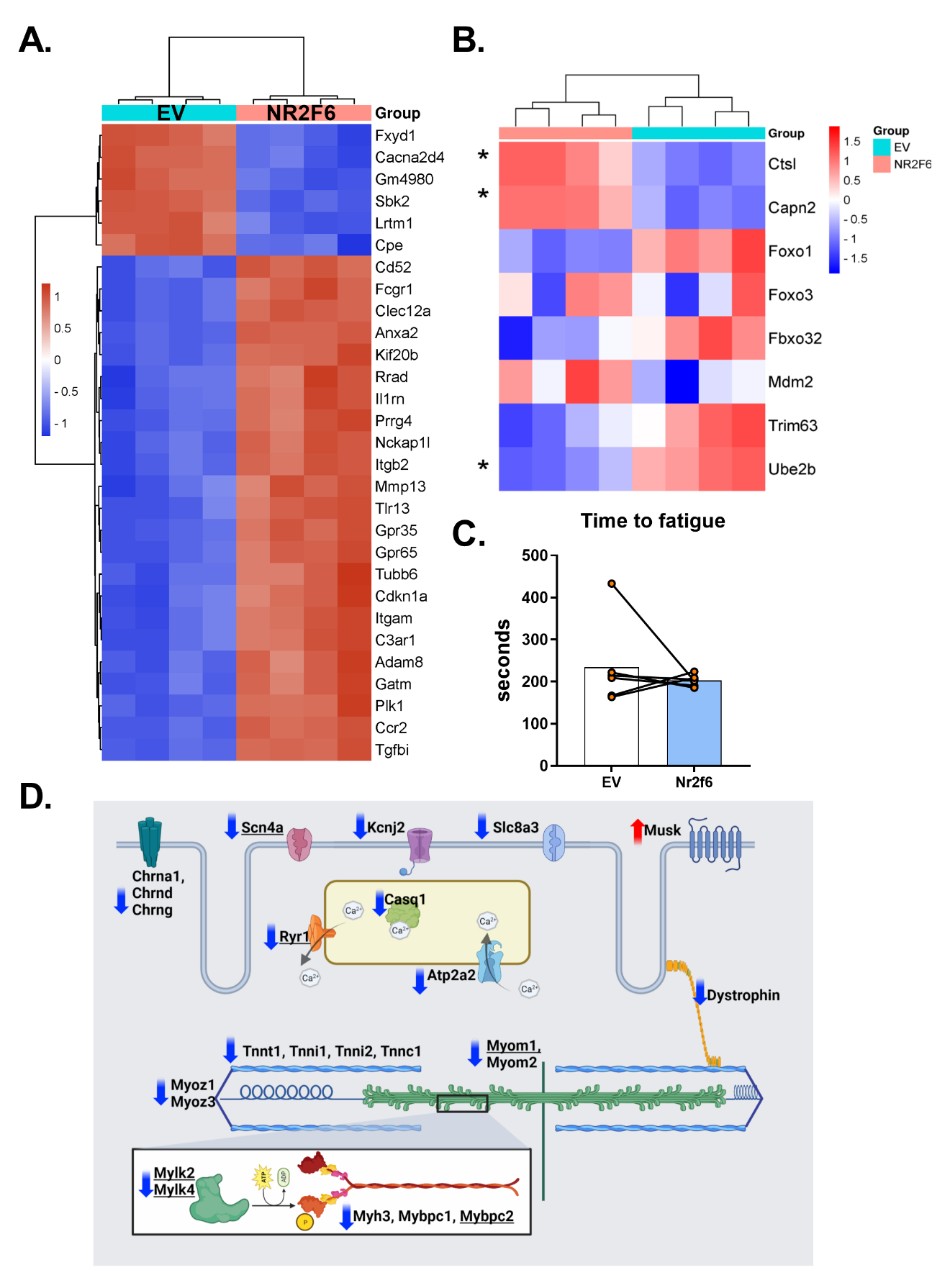


**Fig. S4. Nr2f6 overexpression impairs muscle function.** (A) Heat-map of top 30 most modulated genes in tibialis anterior muscle electroporated with control empty vector (EV) or an Nr2f6-myc-coding plasmid (N = 4). (B) Atrogenes regulated by Nr2f6 overexpression in the tibialis anterior muscle. * Denotes significant modulation (FDR < 0.05, fold-change > 2) in the microarray. (C) Time to fatigue in ex vivo contraction was set as the necessary time to reach 50% of the maximal force with constant stimulation (N = 6). (D) Nr2f6 overexpression reduces the expression of several genes of the contractile apparatus, myofiber calcium handling, and action potential transduction. Genes with Nr2f6 binding motif at the promoter are underscored. Differentially expressed genes following Nr2f6 overexpression in mouse TA were selected according to ontology terms related to muscle contraction and function. The arrows indicate the up- or downregulation. Sodium Voltage-Gated Channel Alpha Subunit 4 (Scn4a), Potassium Inwardly Rectifying Channel Subfamily J Member 2 (Kcnj2), Solute Carrier Family 8 Member A3 (Slc8a3), Muscle Associated Receptor Tyrosine Kinase (Musk), Ryanodine Receptor 1 (Ryr1), Calsequestrin 1 (Casq1), ATPase Sarcoplasmic/Endoplasmic Reticulum Ca2+ Transporting 2 (SERCA2, Atp2a2), Cholinergic Receptor Nicotinic Alpha 1/delta/gamma subunit Chrna1/d/g), Troponin T1/I1/I2/C1 (Tnnt1/Tnni1/Tnni2/c1), Myom1/2 (Myomesin1/2), Myozenin1/3 (Myoz1/3), Myosin light chain kinase 2/4 (Mylk2/4), Myosin heavy chain 3 (Myh3), Myosin binding protein C1/2 (Mybpc1/2).


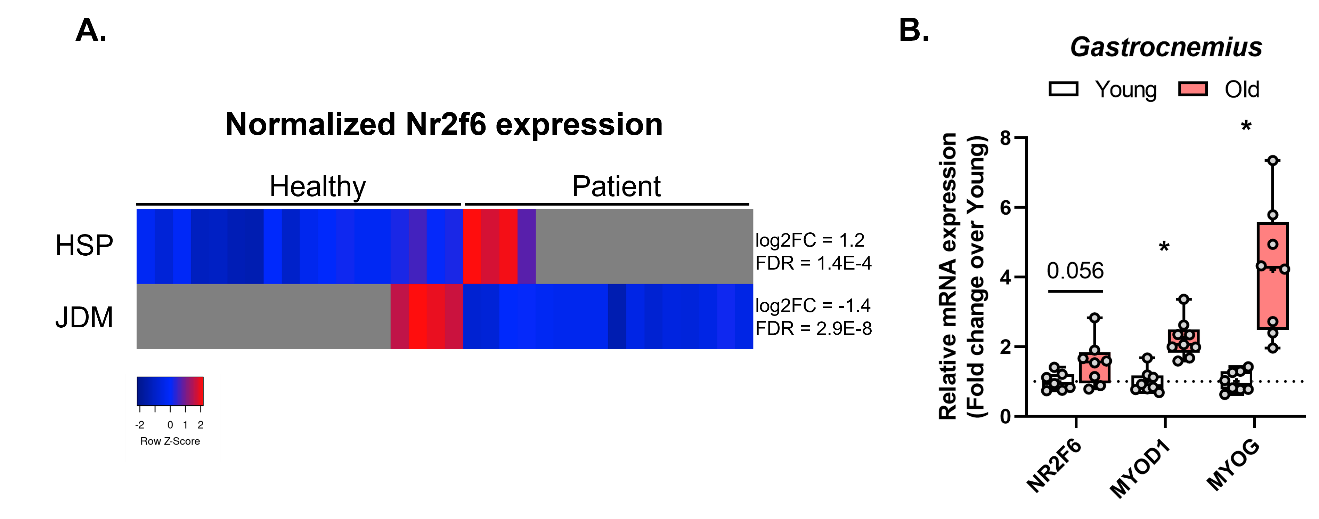


**Fig. S5. Increased Nr2f6 expression in aged muscle. (A) Nr2f6 expression in muscles of healthy donors and bearers of hereditary spastic paraplegia (HSP) and juvenile dermatomyositis (JDM) from public datasets, GSE3307 and** **GSE11971, respectively. (B) Gastrocnemius muscles of 3-month-old (Young) and 18-month-old (Old) mice were collected and processed for RT-qPCR as described elsewhere (N = 7-9). Boxplot with whiskers spanning minimum to maximal and box edges 25th-75th percentile, the line at the median and + symbol at the mean. p-values < 0.05 depicted as *.**

**
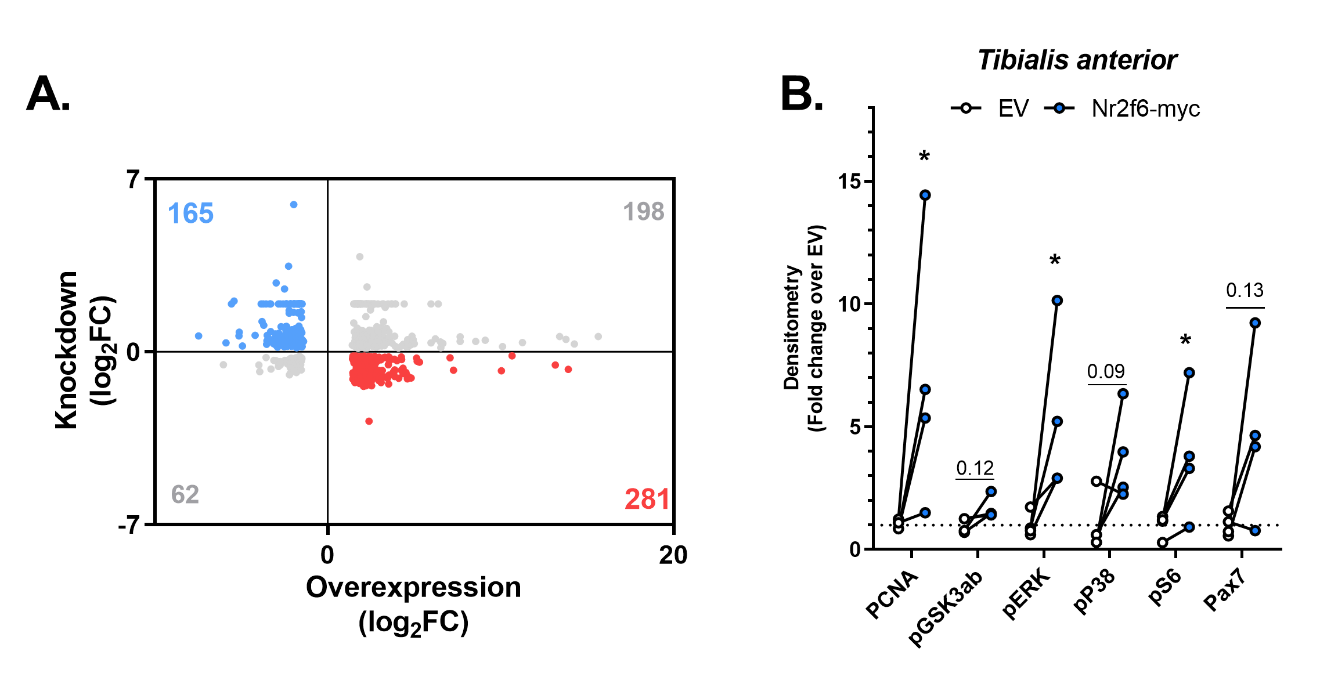
**

**Fig. S6.** (A) Scatter plot of differentially expressed genes in Nr2f6 knockdown in C2C12 myocytes RNA-seq (FDR < 0.05) and Nr2f6 overexpression in mice TA microarray (FDR <0.05, fold-change >2). In red: genes upregulated by Nr2f6; in blue: genes downregulated by Nr2f6; in grey: genes with the same direction of modulation by Nr2f6 overexpression and knockdown. (B) Quantitation of the indicated proteins by western blot in *tibialis anterior* muscles electroporated with empty vector (EV) in the lateral leg or Nr2f6-myc plasmid in the contralateral leg (N = 4). Individual samples are depicted as circles and * indicates p < 0.05 using ratio paired two-tailed Student’s t-tests, when larger than 0.05, p-values are indicated above comparisons.


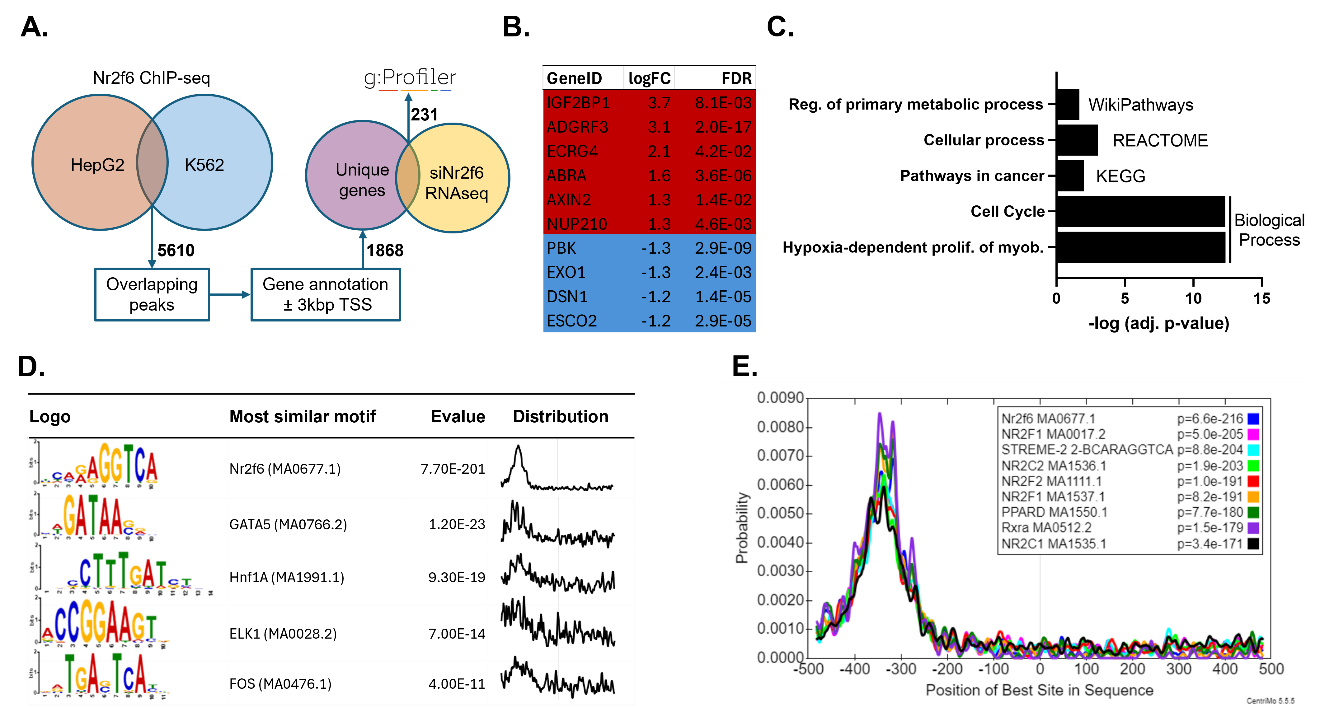


**Fig. S7 Bioinformatic analyses of Nr2f6 ChIP-seq Nr2f6 peak regions from HepG2 and K562 cells were retrieved from the UCSC genome browser, and the overlapping sequences were extracted. *De novo* motif enrichment was performed in the resulting sequences and the corresponding most similar transcription factor motif was attributed. (A) Workflow of bioinformatic analysis of Nr2f6 ChIP-seq data. (B) Top 10 largest fold-change values in siNr2f6 RNA-seq that contain an Nr2f6 binding peak in both ChIP-seq experiments. (C) Gene ontology of the unique genes regulated by Nr2f6 knockdown. (D) De novo motif analysis of Nr2f6 binding regions conserved in both ChIP-seq data. (E) Transcription factors are most likely represented by the lowest E-value matrix.**

**References (continuation)**

S1. Kostrominova TY, Macpherson PCD, Carlson BM, Goldman D. Regulation of myogenin protein expression in denervated muscles from young and old rats. *Am J Physiol - Regul Integr Comp Physiol* 2000

S2. Musarò A, Cusella De Angelis MG, Germani A, Ciccarelli C, Molinaro M, Zani BM. Enhanced expression of myogenic regulatory genes in aging skeletal muscle. *Exp Cell Res* 1995

S3. Bakay M, Wang Z, Melcon G, Schiltz L, Xuan J, Zhao P *et al.* Nuclear envelope dystrophies show a transcriptional fingerprint suggesting disruption of Rb-MyoD pathways in muscle regeneration. *Brain* 2006

S4. Casari G, De Fusco M, Ciarmatori S, Zeviani M, Mora M, Fernandez P *et al.* Spastic paraplegia and OXPHOS impairment caused by mutations in paraplegin, a nuclear-encoded mitochondrial metalloprotease. *Cell* 1998

S5. McDermott CJ, Dayaratne RK, Tomkins J, Lusher ME, Lindsey JC, Johnson MA *et al.* Paraplegin gene analysis in hereditary spastic paraparesis (HSP) pedigrees in northeast England. *Neurology* 2001

S6. Fink JK. Hereditary spastic paraplegia: Clinico-pathologic features and emerging molecular mechanisms. Acta Neuropathol. 2013

S7. Vattemi G, Mirabella M, Guglielmi V, Lucchini M, Tomelleri G, Ghirardello A *et al.* Muscle biopsy features of idiopathic inflammatory myopathies and differential diagnosis. Autoimmun. Highlights. 2014

S8. Núñez L, Buxbaum AR, Katz ZB, Lopez-Jones M, Nwokafor C, Czaplinski K *et al.* Tagged actin mRNA dysregulation in IGF2BP1−/− mice. *Proc Natl Acad Sci U S A* 2022

S9. Elcheva IA, Wood T, Chiarolanzio K, Chim B, Wong M, Singh V *et al.* RNA-binding protein IGF2BP1 maintains leukemia stem cell properties by regulating HOXB4, MYB, and ALDH1A1. *Leukemia* 2020

S10. Xu X, Leng J, Zhang X, Capellini TD, Chen Y, Yang L *et al.* Identification of IGF2BP1-related lncRNA-miRNA-mRNA network in goat skeletal muscle satellite cells. *Anim Sci J* 2021

S11. Sabatier R, Finetti P, Adelaide J, Guille A, Borg JP, Chaffanet M *et al.* Down-regulation of ECRG4, a candidate tumor suppressor gene, in human breast cancer. *PLoS One* 2011

S12. Huang L, Yu H, Fan X, Li X, Mao L, Cheng J *et al.* A potential role of esophageal cancer related gene-4 for atrial fibrillation. *Sci Rep* 2017

S13. Carr JC, Boese EA, Spanheimer PM, Dahdaleh FS, Martin M, Calva D *et al.* Differentiation of small bowel and pancreatic neuroendocrine tumors by gene-expression profiling. *Surg (United States)* 2012

S14. Dietrich DR. Toxicological and pathological applications of proliferating cell nuclear antigen (PCNA), a novel endogenous marker for cell proliferation. *Crit Rev Toxicol* 1993

S15. Pansters NAM, Schols AMWJ, Verhees KJP, de Theije CC, Snepvangers FJ, Kelders MCJM *et al.* Muscle-specific GSK-3β ablation accelerates regeneration of disuse-atrophied skeletal muscle. *Biochim Biophys Acta - Mol Basis Dis* 2015

S16. Ma Z, Zhong Z, Zheng Z, Shi XM, Zhang W. Inhibition of glycogen synthase kinase-3β attenuates glucocorticoid-induced suppression of myogenic differentiation in vitro. *PLoS One* 2014

S17. Michailovici I, Harrington HA, Azogui HH, Yahalom-Ronen Y, Plotnikov A, Ching S *et al.* Nuclear to cytoplasmic shuttling of ERK promotes differentiation of muscle stem/progenitor cells. *Dev* 2014

S18. Jones NC, Fedorov Y V., Rosenthal RS, Olwin BB. ERK1/2 is required for myoblast proliferation but is dispensable for muscle gene expression and cell fusion. *J Cell Physiol* 2001

S19. Yi YW, You K, Jeongbae E, Kwak SJ, Seong YS, Bae I. Dual inhibition of EGFR and MET induces synthetic lethality in triple-negative breast cancer cells through downregulation of ribosomal protein S6. *Int J Oncol* 2015

S20. Ruvinsky I, Meyuhas O. Ribosomal protein S6 phosphorylation: from protein synthesis to cell size. Trends Biochem. Sci. 2006

S21. Hermann-Kleiter N, Gruber T, Lutz-Nicoladoni C, Thuille N, Fresser F, Labi V *et al.* The Nuclear Orphan Receptor NR2F6 Suppresses Lymphocyte Activation and T Helper 17-Dependent Autoimmunity. *Immunity* 2008

S22. Olson WJ, Jakic B, Labi V, Schoeler K, Kind M, Klepsch V *et al.* Orphan Nuclear Receptor NR2F6 Suppresses T Follicular Helper Cell Accumulation through Regulation of IL-21. *Cell Rep* 2019

S23. Yang SL, Guan HQ, Yang HB, Chen Y, Huang XY, Chen L *et al.* The expression and biological effect of NR2F6 in non-small cell lung cancer. *Front Oncol* 2022;**12**:940234.

S24. Avram D, Ishmael JE, Nevrivy DJ, Peterson VJ, Lee SH, Dowell P *et al.* Heterodimeric interactions between chicken ovalbumin upstream promoter- transcription factor family members ARP1 and Ear2. *J Biol Chem* 1999

S25. Huttlin EL, Bruckner RJ, Navarrete-Perea J, Cannon JR, Baltier K, Gebreab F *et al.* Dual proteome-scale networks reveal cell-specific remodeling of the human interactome. *Cell* 2021

S26. Kupr B, Schnyder S, Handschin C. Role of nuclear receptors in exercise-induced muscle adaptations. Cold Spring Harb. Perspect. Med. 2017

S27. Verbrugge SAJ, Schönfelder M, Becker L, Nezhad FY, de Angelis MH, Wackerhage H. Genes whose gain or loss-of-function increases skeletal muscle mass in mice: A systematic literature review. Front. Physiol. 2018

S28. Shimizu N, Maruyama T, Yoshikawa N, Matsumiya R, Ma Y, Ito N *et al.* A muscle-liver-fat signalling axis is essential for central control of adaptive adipose remodelling. *Nat Commun* 2015

S29. Mayeuf-Louchart A, Thorel Q, Delhaye S, Beauchamp J, Duhem C, Danckaert A *et al.* Rev-erb-α regulates atrophy-related genes to control skeletal muscle mass. *Sci Rep* 2017

S30. Yamamoto H, Williams EG, Mouchiroud L, Cantó C, Fan W, Downes M *et al.* NCoR1 is a conserved physiological modulator of muscle mass and oxidative function. *Cell* 2011;**147**:827–839.

S31. Lee H-J, Kao C-Y, Lin S-C, Xu M, Xie X, Tsai SY *et al.* Dysregulation of nuclear receptor COUP-TFII impairs skeletal muscle development OPEN.

S32. Xie X, Tsai SY, Tsai MJ. COUP-TFII regulates satellite cell function and muscular dystrophy. *J Clin Invest* 2016

S33. Pearen MA, Eriksson NA, Fitzsimmons RL, Goode JM, Martel N, Andrikopoulos S *et al.* The nuclear receptor, Nor-1, markedly increases type II oxidative muscle fibers and resistance to fatigue. *Mol Endocrinol* 2012

S34. Chen W, Zhang X, Birsoy K, Roeder RG. A muscle-specific knockout implicates nuclear receptor coactivator MED1 in the regulation of glucose and energy metabolism. *Proc Natl Acad Sci U S A* 2010

S35. Seth A, Steel JH, Nichol D, Pocock V, Kumaran MK, Fritah A *et al.* The Transcriptional Corepressor RIP140 Regulates Oxidative Metabolism in Skeletal Muscle. *Cell Metab* 2007

S36. Son C, Hosoda K, Ishihara K, Bevilacqua L, Masuzaki H, Fushiki T *et al.* Reduction of diet-induced obesity in transgenic mice overexpressing uncoupling protein 3 in skeletal muscle. *Diabetologia* 2004

S37. Darcy MacLellan J, Gerrits MF, Gowing A, Smith PJS, Wheeler MB, Harper ME. Physiological increases in uncoupling protein 3 augment fatty acid oxidation and decrease reactive oxygen species production without uncoupling respiration in muscle cells. *Diabetes* 2005

S38. Clapham JC, Arch JRS, Chapman H, Haynes A, Lister C, Moore GBT *et al.* Mice overexpressing human uncoupling protein-3 in skeletal muscle are hyperphagic and lean. *Nature* 2000

S39. Son C, Hosoda K, Matsuda J, Fujikura J, Yonemitsu S, Iwakura H *et al.* Up-regulation of uncoupling protein 3 gene expression by fatty acids and agonists for PPARs in L6 myotubes. *Endocrinology* 2001

S40. Weigle DS, Selfridge LE, Schwartz MW, Seeley RJ, Cummings DE, Havel PJ *et al.* Elevated free fatty acids induce uncoupling protein 3 expression in muscle: A potential explanation for the effect of fasting. *Diabetes* 1998

S41. Cruz-Jentoft AJ, Sayer AA. Sarcopenia. Lancet. 2019

S42. Picca A, Calvani R, Bossola M, Allocca E, Menghi A, Pesce V *et al.* Update on mitochondria and muscle aging: All wrong roads lead to sarcopenia. Biol. Chem. 2018

S43. Alexandre PA, Naval-Sanchez M, Porto-Neto LR, Ferraz JBS, Reverter A, Fukumasu H. Systems biology reveals NR2F6 and TGFB1 as key regulators of feed efficiency in beef cattle. *Front Genet* 2019

S44. Klepsch V, Gerner RR, Klepsch S, Olson WJ, Tilg H, Moschen AR *et al.* Nuclear orphan receptor NR2F6 as a safeguard against experimental murine colitis. *Gut* 2018

S45. Pelletier L, Petiot A, Brocard J, Giannesini B, Giovannini D, Sanchez C *et al.* In vivo RyR1 reduction in muscle triggers a core-like myopathy. *Acta Neuropathol Commun* 2020

S46. Sakakibara I, Yanagihara Y, Himori K, Yamada T, Sakai H, Sawada Y *et al.* Myofiber androgen receptor increases muscle strength mediated by a skeletal muscle splicing variant of Mylk4. *iScience* 2021

S47. Lee NKL, Maclean HE. Polyamines, androgens, and skeletal muscle hypertrophy. J. Cell. Physiol. 2011

S48. Cervelli M, Leonetti A, Duranti G, Sabatini S, Ceci R, Mariottini P. Skeletal Muscle Pathophysiology: The Emerging Role of Spermine Oxidase and Spermidine. *Med Sci* 2018

S49. Wang L, Long H, Zheng Q, Bo X, Xiao X, Li B. Circular RNA circRHOT1 promotes hepatocellular carcinoma progression by initiation of NR2F6 expression. *Mol Cancer* 2019

S50. Liu J, Li T, Liu XL. DDA1 is induced by NR2F6 in ovarian cancer and predicts poor survival outcome. *Eur Rev Med Pharmacol Sci* 2017.

S51. Weatherford ET, Liu X, Sigmund CD. Regulation of renin expression by the orphan nuclear receptors Nr2f2 and Nr2f6. *Am J Physiol - Ren Physiol* 2012

S52. Chu K, Zingg HH. The nuclear orphan receptors COUP-TFII and Ear-2 act as silencers of the human oxytocin gene promoter. *J Mol Endocrinol* 1997

S53. Jin C, Xiao L, Zhou Z, Zhu Y, Tian G, Ren S. MiR-142-3p suppresses the proliferation, migration and invasion through inhibition of NR2F6 in lung adenocarcinoma. *Hum Cell* 2019

S54. Raichur S, Lau P, Staels B, Muscat GEO. Retinoid-related orphan receptor γ regulates several genes that control metabolism in skeletal muscle cells: Links to modulation of reactive oxygen species production. *J Mol Endocrinol* 2007

S55. Iqbal J, Jahangir Z, Veluru D, Otaibi A Al, Mubarak SA, Subie B Al *et al.* Deletion of retinoic acid-related orphan receptor gamma reduces body weight and hepatic lipids in mice by modulating the expression of lipid metabolism genes. *Vessel Plus* 2019

S56. Fang Y, Yu H, Liang X, Xu J, Cai X. Chk1-induced CCNB1 overexpression promotes cell proliferation and tumor growth in human colorectal cancer. *Cancer Biol Ther* 2014

S57. Huang V, Place RF, Portnoy V, Wang J, Qi Z, Jia Z *et al.* Upregulation of Cyclin B1 by miRNA and its implications in cancer. *Nucleic Acids Res* 2012

S58. Bao B, Yu X, Zheng W. MiR-139-5p Targeting CCNB1 Modulates Proliferation, Migration, Invasion and Cell Cycle in Lung Adenocarcinoma. *Mol Biotechnol* 2022

S59. Rao VK, Ow JR, Shankar SR, Bharathy N, Manikandan J, Wang Y *et al.* G9a promotes proliferation and inhibits cell cycle exit during myogenic differentiation. *Nucleic Acids Res* 2016

S60. Knight JDR, Kothary R. The myogenic kinome: Protein kinases critical to mammalian skeletal myogenesis. Skelet. Muscle. 2011

S61. Beltran-Alvarez P, Pérez-Villa F, Garcia-Bassets I, Pérez-Serra A, Brugada R, Beltran-Alvarez P *et al.* Transcriptional regulation of the sodium channel gene (SCN5A) by GATA4 in human heart. *J Mol Cell Cardiol* 2017

S62. Afouda BA. Towards Understanding the Gene-Specific Roles of GATA Factors in Heart Development: Does GATA4 Lead the Way? Int. J. Mol. Sci. 2022

S63. Boyer JG, Prasad V, Song T, Lee D, Fu X, Grimes KM *et al.* ERK1/2 signaling induces skeletal muscle slow fiber-type switching and reduces muscular dystrophy disease severity. *JCI Insight* 2019

S64. Khurana A, Dey CS. Involvement of Elk-1 in L6E9 skeletal muscle differentiation. *FEBS Lett* 2002

S65. Li SZ, Zhang ZY, Chen J, Dong MY, Du XH, Gao J *et al.* NLK is required for Ras/ERK/SRF/ELK signaling to tune skeletal muscle development by phosphorylating SRF and antagonizing the SRF/MKL pathway. *Cell Death Discov* 2022

S66. Almada AE, Horwitz N, Price FD, Gonzalez AE, Ko M, Bolukbasi OV *et al.* FOS licenses early events in stem cell activation driving skeletal muscle regeneration. *Cell Rep* 2021

S67. Barutcu AR, Elizalde G, Gonzalez AE, Soni K, Rinn JL, Wagers AJ *et al.* Prolonged FOS activity disrupts a global myogenic transcriptional program by altering 3D chromatin architecture in primary muscle progenitor cells. *Skelet Muscle* 2022

S68. Li X, Wang W, Wang J, Malovannaya A, Xi Y, Li W *et al.* Proteomic analyses reveal distinct chromatin‐associated and soluble transcription factor complexes. *Mol Syst Biol* 2015

S69. Tajsharghi H, Oldfors A. Myosinopathies: Pathology and mechanisms. Acta Neuropathol. 2013

S70. Schiaffino S. Muscle fiber type diversity revealed by anti-myosin heavy chain antibodies. FEBS J. 2018
